# Experimental Elucidation of a Cubane Water Cluster in the Hydrophobic Cavity of UiO‐66

**DOI:** 10.1002/cphc.202400583

**Published:** 2024-10-27

**Authors:** Kazutaka Sonobe, Satoshi Tominaka, Akihiko Machida

**Affiliations:** ^1^ Center for Basic Research on Materials National Institute for Materials Science Tsukuba Ibaraki 305–0044 Japan; ^2^ Synchrotron Radiation Research Center National Institutes for Quantum Science and Technology (QST) SPring-8 Sayo, Hyogo 679-5148 Japan

**Keywords:** Cubane water cluster, Hydrogen bonds, Hydrophobic nanocage, Metal-organic frameworks, Nanotechnology

## Abstract

Nanoscale water plays a pivotal role in determining the properties and functionalities of materials, and the precise control of its quantity and atomic‐scale ordered structure is a focal point in nanotechnology and chemistry. Several studies have theoretically discussed the nano‐ordered ice within one‐ or two‐dimensional space and without confinement through hydrogen bonds. In particular, the water cluster has been predicted to play a significant role in biomolecules or functional nanomaterials; however, there has been little experimental evidence for their presence in hydrophobic cavities. In this study, the cubane water octamer – the most stable isomer among small water clusters – was detected within the hydrophobic cavities of UiO‐66 metal–organic frameworks, revealing the presence of the smallest ice in their hydrophobic cavity, in the absence of hydrogen bonding. This observation contrasts earlier examples of water clusters confined within nanocavities through hydrogen bonds and provides experimental evidence for water‐cluster capturing within hydrophobic cavities. Consequently, our renewed understanding of hydrophilicity and hydrophobicity warrants a design re‐evaluation of materials for chemical applications, including fuel cells, water harvesting, catalysts, and batteries.

## Graphical abstract

This work highlights two intuitively different hydrophobicity between macroscopic and nanoscale. The adsorption of water cluster on hydrophobic nanocage without linked to host materials by hydrogen bonds was elucidated experimentally, although hydrophobicity is regarded as repellent behavior in macroscale. This nanoscale water behavior proposes necessary of reconsideration for design of hydrophobicity and hydrophilicity of functional materials.

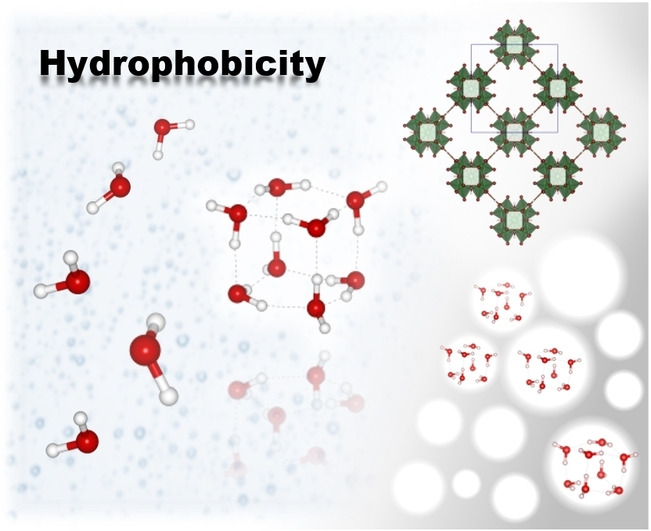

## Introduction

**Figure 1:**
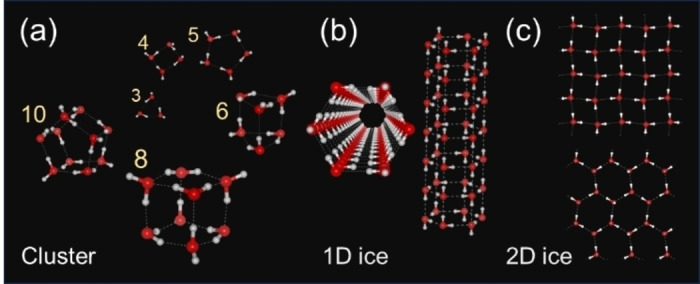
Structure of a low‐dimensional ordered water isomer. (a) 0D water‐cluster structures predicted by ab initio calculations. (b, c) Reported 1D and 2D structures in the hydrophobic spaces of carbon nanotubes and graphene.

Nanotechnology has reached a pivotal stage where the precise control over the presence and transport of water beyond the implicit chemistry that governs processes such as protein folding[^1^, ^2^, ^3^] and low‐dimensional fluid dynamics is essential.[^4^, ^5^] For example, although the role of water in facilitating proton transport is critical for catalysis in fuel cells,[^6^, ^7^] water can also obstruct the pathways necessary for oxygen and hydrogen to reach the catalysts. In addition, the presence of a few water molecules in nanochannels can significantly accelerate thermal catalytic reactions^[8]^ and molecular transportation.[^9^, ^10^] To design materials with reliable and reproducible properties, the presence and behavior of water must be meticulously controlled at the atomic level. Crucially, in material design, hydrophilicity and hydrophobicity are often considered as a water‐attracting or ‐repelling behavior on a macroscopic interface; however, this is not true at the nanoscale. For instance, one‐ or two‐dimensional ice formations in hydrophobic nanospaces, such as graphene[^11^, ^12^] or carbon nanotube cavities,[^10^, ^13^, ^14^] are considered exceptional cases (Figure 1). However, the experimental evidence of water clusters in hydrophobic channels or more typically narrow cavities has not been revealed in atomic resolutions.

From a classical thermodynamic perspective, water molecules in bulk liquid water benefit from the stabilizing effects of strong hydrogen bonding (around 0.46 eV per molecule)^[15]^ and the high entropy of the liquid state. Therefore, to confine a water molecule within a cavity, a stronger attractive force is required to overcome these stabilizing factors. Traditional views suggest that van der Waals forces and other non‐bonding interactions are simply too weak to achieve this confinement in nanoscale cavities.[^10^, ^11^, ^12^, ^13^, ^14^] In such cases, the presence of water within hydrophobic cavities might be explained by the formation of clusters that lack the extensive hydrogen bonding network observed in bulk liquid water. Unlike bulk water, where hydrogen bonds create extensive networks, water clusters are stabilized by closed‐chain hydrogen‐bonding configurations,^[16]^ enabling them to act independently of external materials and similarly to macromolecules. Larger clusters, such as water octamers, have sufficient internal hydrogen bonds that make them as stable as bulk water. This is evidenced by the detection of water clusters within cavities of organic molecules[^17^, ^18^, ^19^, ^20^, ^21^] and proteins;[^22^, ^23^, ^24^] these are often anchored to adjacent functional groups by hydrogen bonds containing heteroatoms such as oxygen and nitrogen, making the cavities partially hydrophilic. However, the observation of water clusters not stabilized by hydrogen bonding in hydrophobic environments is rare.

In this study, we present the first experimental observation of an ordered water octamer within the hydrophobic nanocavity of a UiO‐66 metal‐organic framework (MOF) at room temperature. Previous literature on confined water clusters in MOFs[^18^, ^25^] and organic porous materials[^19^, ^20^] has shown strong interactions via hydrogen bonds with hydrophilic heteroatoms. On the contrary, our findings suggest that water clusters can even be stabilized by weak interactions, thus eliminating the need for strong hydrogen bonds with the framework. This discovery challenges macroscopic intuition for hydrophobicity and has significant implications for material design in various chemical applications.

To analyze water molecules within nanopores in detail, we employed the host–guest interactions offered by MOFs. UiO‐66 is a well‐studied MOF system composed of zirconium oxide clusters (Zr_6_O_4_(OH)_4_) linked by terephthalate ligands.^[26]^ This structure creates an MOF that remains stable upon hydration and boasts a high surface area with both hydrophobic and hydrophilic environments, making it ideal for investigating water behavior in nanoscale spaces. Unlike many other MOFs that degrade upon hydration, UiO‐66 is renowned for its robustness.[^27^, ^28^, ^29^] Although host–guest interactions – including water‐framework interactions within UiO‐66 – have been previously reported^[30]^ and are common with MOFs,[^31^, ^32^] there remains scope for further exploration of water molecules within this framework. This is because the X‐ray structural analyses of MOFs typically involve removing guest molecules as much as possible to elucidate the structure of the host framework. In contrast, our focus is on the structure and state of the guest water molecules, similar to the crystalline sponge method to focus guest material,[^33^, ^34^] particularly those residing within the hydrophobic pores.

## Results and Discussion

UiO‐66 samples were synthesized following established procedures described in the literature.[^27^, ^35^] The quality of the samples was confirmed using standard characterization techniques, such as powder diffraction. Full details on the synthesis and characterization can be found in the supporting information. We prepared UiO‐66 powders with varying degrees of hydration. Briefly, the synthesized crystalline powders were activated by vacuum treatment (Figure S1) to remove guest molecules from the pores. Subsequently, the samples were exposed to ambient atmosphere for water uptake (hydrated state), partially dehydrated at 100 °C for 12 h, and fully dehydrated at 100 °C for 3 d. Thermogravimetric analysis (TGA) was employed to determine the framework composition, including linker molecule deficiencies, for the hydrated sample. The analysis revealed the formula [Zr_6_O_4_OH_4_(bdc)_5.28_(ac)_1.44_] (bdc=C_6_H_4_(COO)_2_, ac=CH_3_COO), which is consistent with the presence of residual acetate ligands at linker‐deficient sites based on the TGA results. The TGA also identified two distinct types of water molecules within the framework: weakly adsorbed water molecules desorbed at up to approximately 63 °C (8.77 molecules per framework unit) and strongly adsorbed water molecules desorbed at up to approximately 144 °C (4.0 molecules per framework unit).

**Figure 2:**
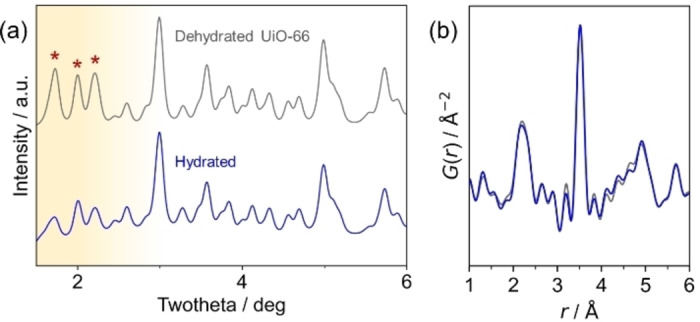
Insertion of ordered water into hydrophobic spaces. (a) X‐ray diffraction patterns of UiO‐66 during dehydration. The differences in the diffraction intensity, which reveal the effect of the water content, are indicated with asterisks. (b) Pair distribution functions of the same data, revealing the ordered structure of water within the nanospaces of UiO‐66.

Experimentally observing water clusters within nanopores presents a challenge – guest water molecules can either occupy well‐defined, crystallographically symmetric sites, identifiable through X‐ray crystallography, or adopt a more disordered, amorphous arrangement. To account for both scenarios in our investigation, we employed high‐energy synchrotron X‐ray scattering, which offers two key advantages – it provides reciprocal space data suitable for crystallographic analysis (Figure 2a); and, real‐space data through Fourier transformation (Figure 2b) for a comprehensive understanding of the structure, including the distribution of amorphous water molecules.^[36]^ Furthermore, using the high‐angle region of the reciprocal space data provides higher resolution in real‐space analysis. However, a trade‐off exists – high‐energy X‐rays offer lower resolution in reciprocal space, hindering phase determination. Therefore, we characterized the synthesized UiO‐66 using powder diffraction with Cu Kα radiation prior to the synchrotron experiments (details in the supporting information). As shown in Figure S2, the hydration levels significantly impact the lattice vibrations or symmetry. These structural changes induced by hydration are discussed in the following paragraphs.

Rietveld analysis was used to characterize the fully dehydrated UiO‐66 as a cubic phase (space group: *F* $\bar{4}$ 3 *m*, *a*=20.75497(14) Å, weighted *R* factor (*R_wp_*)=2.33 %) (Figure S4) exhibiting lower symmetry than typically reported (*Fm* $\bar{3}$ *m*) for pristine samples, which can be attributed to polyhedral distortion and proton localization within the Zr_6_O_8_H_4_ clusters (*O*_h_‐to‐*T*_h_ point group changes).[^37^, ^38^] The UiO‐66 structure comprises two distinct pore types, octahedral and tetrahedral nanocages, and we focused on the latter. These smaller cavities, which host water molecules, were further categorized based on the presence of OH groups within the Zr_6_O_4_(OH)_4_ units.

Interestingly, the hydration caused subtle changes in peak intensities, hinting at water molecules entering the dehydrated crystal structure. The hydrated sample that was prepared under 60 % RH humidity level at 20 °C, where the water adsorption saturates according to reported vapor water isotherms,^[30]^ was compared with a dried sample. The structural difference was reflected in the XRD patterns, particularly at lower angles (Figure 2a). Notably, these changes were not due to framework decomposition, as confirmed by the PDFs (Figures 2b and S3). This suggests the formation of an ordered water structure within the UiO‐66 nanoscale pores.

**Figure 3:**
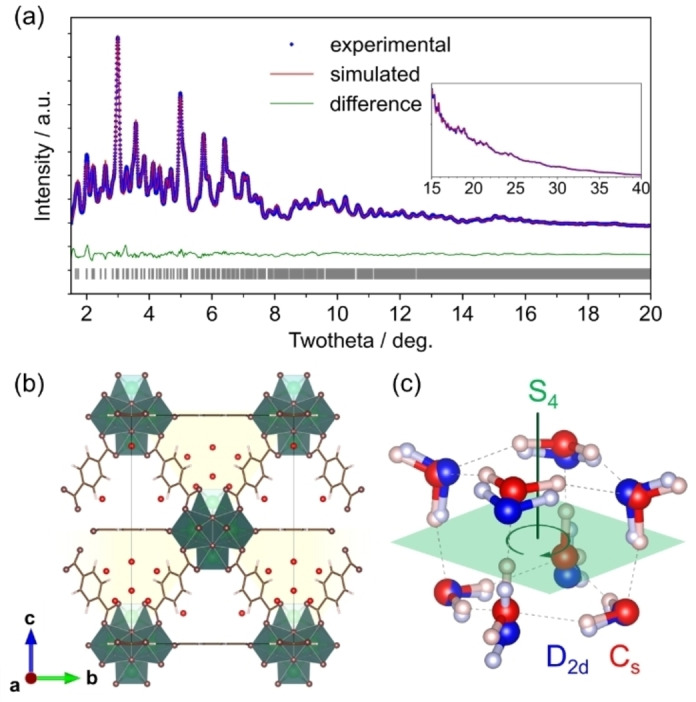
Determination of the crystal structure of the water octamer within UiO‐66. (a) Rietveld fitting of the observed X‐ray diffraction pattern for hydrated UiO‐66. The inset shows fitting at higher angles. (b) Derived crystal structural model, highlighting the hydrophobic cage in yellow. (c) Stable structure of the water octamer within hydrophobic cages (red) and that outside cage (calculation in vacuum) (blue). Here, the hydrogen atoms were added based on the most stable positions of the cubane octamer. Notably, the *S*_4_ symmetry observed for the water octamer in a vacuum is disrupted within the hydrophobic cage.

Subsequent water sorption led to noticeable changes in peak intensities, and Rietveld refinement identified the hydrated UiO‐66 as an orthorhombic structure (space group: *Imm*2, *a*=14.6729(2) Å, *b*=14.6729(2) Å, *c*=20.7506(3) Å, *R_wp_* =3.82 %) (Figures 3a and b). In addition to the four water molecules in the OH‐equipped tetrahedral pores, eight more molecules were found within the remaining tetrahedral nanocages, reducing the overall crystal symmetry. Similarly, the amount of water was the same as that of the weakly adsorbed water measured by thermogravimetry (Figure S6). This distribution of water molecules is consistent with the reported stepwise dehydration behavior of UiO‐66.[^39^, ^40^] The oxygen‐to‐oxygen distances, which were larger than 3.9 Å, eliminate the possibility of hydrogen bonding with the zirconia cluster, thus indicating other stabilization mechanisms for these molecules. Compared with the known water clusters bound to the MOF nanocavities lining via hydrogen bonds,[^20^, ^25^] this octamer lacks such strong hydrogen bonds because of the absence of nearby heteroatoms suitable for bonding, as observed in the nanospaces of nanocarbons (Figure S9).

Within the tetrahedral cages, the eight water molecules assemble into isolated octamers, forming a cubane structure stabilized by three hydrogen bonds (Figure 3c) for a water molecule, which is comparable to the mean number of hydrogen bonds of liquid water (2.2–3.4 bonds).^[41]^ Vibration rotation tunneling (VRT) spectroscopy study have reported several isomers of this water octamer,^[42]^ and the one with *D*_2d_ symmetry was the equilibrium configuration for the cubane cluster. Further, our findings revealed that the octamer within UiO‐66 undergoes a distortion (point group: *C*_s_), breaking the improper rotation symmetry (*S*_4_) in the *D*_2d_ point group. This distortion, coupled with the positioning of water oxygen atoms at symmetry locations within the framework, suggests interactions with the UiO‐66 interior surface. The cubane octamer is recognized as the most stable water cluster composed of fewer than ten water molecules, and its structural variations arising from the changes in proton configurations and hydrogen bonds, which result in multiple stable structures, have been well documented.[^43^, ^44^, ^45^]

**Figure 4:**
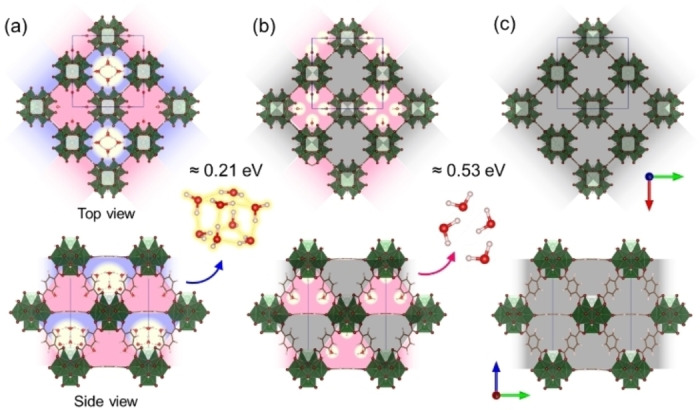
Crystal structure models (top: ab plane, bottom: bc plane) illustrating the stepwise desorption of water molecules from UiO‐66. The structures represent the (a) hydrated, (b) partially hydrated, and (c) dehydrated states. Hydrophobic and hydrophilic cages are depicted in blue and pink, respectively, indicating the presence of water molecules. Empty cages are shown in gray. Quantum chemical simulations predicted that weakly adsorbed water molecules, identified by X‐ray analysis within the hydrophobic cages, possess a lower adsorption energy of approximately 0.21 eV. In contrast, the stably adsorbed water molecules found in hydrophilic cages exhibit a higher adsorption energy of approximately 0.53 eV.

The adsorption energies of water on hydrophilic and hydrophobic cavities in several hydration states were compared (Figure 4). In addition to the hydrated model discovered in this work, the partially hydrated model (Figure 4b), which has been revealed in a previous work, was observed. The partially hydrated sample maintained a similar cubic crystal structure (space group: *F* $\bar{4}$ 3 *m*, *a*=20.75664(17) Å, *R_wp_* =2.59 %) (Figure S5) but contained four H_2_O molecules[^30^, ^36^, ^46^] in the tetrahedral pore with OH groups. The amount of water was the same as that of the strongly adsorbed water measured by thermogravimetry (Figure S6). The distance between the oxygen atoms of water and the zirconia cluster was 2.8 Å, which is typical^[47]^ for hydrogen‐bonded oxygen‐to‐oxygen distances. Thus, the UiO‐66 can take at least three different hydration states.

The structural models for the three adsorption states were corroborated by density functional theory (DFT) calculations (Figures 4 and S7), which confirmed the structures energetic favorability, and were validated through linear‐scaling DFT with the VdW‐DF‐optB88 functional. This functional adeptly capture the non‐bonded dispersion interactions between the water octamer and UiO‐66. Before performing calculations on the UiO‐66 model, the cubane structure with a regular octahedral shape was studied using various theoretical methods, including DFT (ωB97X‐D3), the random phase approximation (RPA), and spin‐component‐scaled second‐order Møller–Plesset perturbation theory (SCS‐MP2). The results were consistent across these different theoretical methods, revealing that the observed distortions were intrinsic to the framework and not computational artifacts (Figure S8).

**Figure 5:**
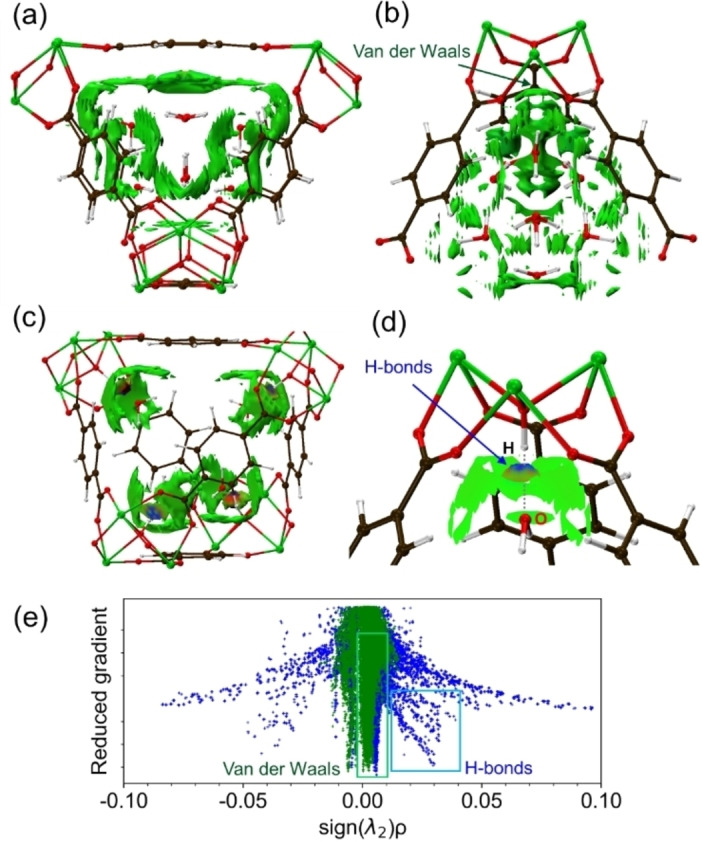
NCI analysis of water adsorption on UiO‐66. (a, b) Van der Waals (VdW) interaction surfaces between the water octamer and nanocage are shown in green. (c, d) Hydrogen bond interaction surfaces between water and the OH groups of Zr_6_O_8_H_4_ are shown in blue and red. (e) Reduced density gradient NCI plot corresponding to the interactions in panels “a” to “d”, where the differences in interaction strength at each coordination in crystal is shown as the difference between value of sign(λ_2_) ρ.

The weak interactions between the water octamer and UiO‐66 framework could be observed through reduced electron density maps obtained from non‐covalent interaction (NCI) analyses (Figure 5).^[48]^ These analyses confirmed the hydrogen bonds between the four water molecules (as mentioned earlier) and the OH groups of the zirconia cluster (Figure 4a), contributing approximately 0.53 eV per molecule to the desorption energy (Figure 4b). The presence of these interactions was further supported by peaks in the reduced density gradient plots (Figure 5e). Concerning the water octamer, the interaction zone extended across the entire tetrahedral cavity wall, indicating that van der Waals forces are the primary driver of adsorption. Furthermore, desorption energy calculations estimated that desorbing the octamer as a whole requires approximately 0.21 eV per molecule, whereas desorbing individual water molecules requires 0.63 eV per molecule (Figure 4a). Notably, considering the evaporation energy of liquid water (0.46 eV), the formed octamer within the cavity might exhibit comparable stability (although the accuracy of simulations may not perfectly match measured heat values). Additionally, NCI analysis suggested a possible secondary role played by weak OH−π interactions with the phenylene rings of UiO‐66, similar to the interactions observed in cubane‐benzene clusters.^[49]^

Moreover, these findings align with the thermogravimetric results (Figure S6), which demonstrated that the octamer evaporates at a lower temperature than liquid water (<63 °C).^[50]^ The TGA analysis reveals a distinct, sharp step for the desorption of cubane water, preceding the gradual desorption of hydrogen‐bonded water. It's worth noting that water desorption from the grain surface often appears as a gradual loss below 100 °C. Consequently, the calculated weaker adsorption energy of water octamers is consistent with the experimental TGA data. Both theoretical calculations and experimental results thus support the evidence for the presence of water octamers within the cavities.

## Conclusions

The crystallographic analysis in this study revealed the first stable water octamer within a hydrophobic nanocavity that was not anchored by strong hydrogen bonds. This discovery highlighted the water cluster behavior, different from the macroscopic view of hydrophobicity, as a repelling behavior; moreover, the typical synthetic view emphasized the necessity of a polar functional group for water to capture water. Further, we demonstrated that water molecules can infiltrate extremely narrow hydrophobic nanospaces, even when larger pores remain unoccupied, without interacting with heteroatoms. These findings emphasize the significant role of cluster chemistry, indicating that the fundamental unit influencing the chemical properties (even for well‐known molecules such as water) may extend beyond the individual molecule. This insight paves the way for an advanced nanospace design, thereby enabling the manipulation of water molecules at the nano‐ and molecular scales and fostering innovation in fields such as fuel cells, water harvesting, catalysts, and batteries by dispelling misconceptions about hydrophobicity.

## Conflict of Interests

The authors declare no conflict of interest.

1

## Supporting information

As a service to our authors and readers, this journal provides supporting information supplied by the authors. Such materials are peer reviewed and may be re‐organized for online delivery, but are not copy‐edited or typeset. Technical support issues arising from supporting information (other than missing files) should be addressed to the authors.

Supporting Information

## Data Availability

We will deposit the data in MRD (a public database in our institute, NIMS) when this paper got accepted.
