# Supplementary material for: Experimental Elucidation of a Cubane Water Cluster in the Hydrophobic Cavity of UiO‐66
Source: Chemphyschem. 2024 Oct 27;25(23):e202400583. doi: 10.1002/cphc.202400583 (PMC11614365; doi:10.1002/cphc.202400583)
Supplement: Supplementary file 1 — Supporting Information [file CPHC-25-e202400583-s001.pdf]

ChemPhysChem

Supporting Information

Experimental Elucidation of a Cubane Water Cluster in the Hydrophobic Cavity of UiO-66

Kazutaka Sonobe, Satoshi Tominaka,* and Akihiko Machida

Supporting Information:

Experimental elucidation of a cubane water cluster in the hydrophobic cavity of UiO-66

Kazutaka Sonobe[1], *Satoshi Tominaka*[1], *Akihiko Machida*[2]

[1]Center for Basic Research on Materials, National Institute for Materials Science, Tsukuba, Ibaraki 305-0044, Japan
[2]Synchrotron Radiation Research Center, National Institutes for Quantum Science and Technology (QST), SPring-8, Sayo, Hyogo 679-5148, Japan

E-mail: TOMINAKA.Satoshi@nims.go.jp

This PDF file includes:

1. Experimental and analysis method
2. Figure S1 to S12
3. Reference
4. Structural data
5. Refinement detail for Rietveld analysis

Experimental and analysis method

Chemicals. Zirconium(Ⅳ) propoxide in 1-propanol (c.a. 70 wt%) was obtained from Tokyo Chemical Industry Co. 1,4-Benzenedicarboxylic acid (bdc), acetic acid, and *N*,*N*- dimethylformamide were obtained from Fujifilm Wako Chemicals Co.

Synthesis of UiO-66. The synthesis of UiO-66 was conducted using the secondary building unit approach. Briefly, zirconium(Ⅳ) propoxide in 1-propanol (70 wt%, 2.0 mL) and acetic acid (3.1 mL) were mixed at 25 °C for 3 days. The generated white precipitate was separated by centrifugation at 4000 rpm for 2 h and dried at 50 °C under vacuum. The white precipitate (200 mg) was then added to acetic acid (5.0 mL). The mixture in acetic acid was heated with a microwave (Biotage Initiator+8) at 200 °C for 1 h and allowed to stand at 25 °C overnight. The powdered zirconium oxide clusters were separated by centrifugation at 4000 rpm for 2 h. Then, the zirconium oxide cluster powder (200 mg) and terephthalic acid (431 mg) were added to a mixture of acetic acid (1.8 mg) and *N*,*N*-dimethylformamide (3.15 mg), and reacted with a microwave at 130 °C for 3 h. The reaction yielded a white precipitate containing UiO-66. The reactant was centrifugated at 4000 rpm overnight, and the precipitate separated into double layers. The upper and lower layers of the precipitate are purified UiO-66 and amorphous byproducts, respectively. The purified sample was activated by vacuum treatment at 100 °C overnight to remove solvents from the pores as confirmed in Figure S1. Three samples with varying degrees of hydration were prepared from this purified UiO-66: (i) Hydrated sample: Prepared by exposing the dried sample to a controlled humidity environment at 20 °C and 60% RH by the advanced air conditioning system to accomplish water uptake.[1] (ii) Partially dehydrated sample: Prepared by drying the hydrated sample at 100 °C for 12 hours. (iii) Fully dried sample: Prepared by drying the hydrated sample at 100 °C for three days

Phase purity of the synthesized UiO-66 was verified using powder X-ray diffractometry (PXRD, Figure S1a) on a Rigaku MiniFlex instrument in Bragg–Brentano geometry with Cu Kα radiation. Pawley refinement yielded a good match between the calculated and experimental PXRD patterns, and the experimental pattern itself was identical to the previously reported diffraction pattern. [2] The removal of solvent molecules from the pores, characteristic of UiO-66, was confirmed by N2 adsorption isotherms measured on a MicrotracBEL BELSORP MINI X instrument. Attenuated total reflectance (ATR) Fourier transform infrared (FTIR) spectra acquired on a Bruker ALPHA II spectrometer further corroborated this finding.

Thermogravimetric analysis (TGA) was employed to determine the content of linker molecules. The analysis was performed on a SIMADZU DTG-60 instrument under air flow (100 ml/sec) with a heating rate of 10°C/min using an alumina pan. An exothermic weight loss of (i) 47.95 wt% was observed. TGA was also employed to quantify the content of guest molecules, this time using a TA Instruments SDT-Q600 instrument under argon gas flow (100 ml/sec) with a heating rate of 1°C/min and an aluminum pan. The differential thermal analysis (DTA) curve was obtained by similar conditions changing the heating rate conditions of the TGA measurement to 10°C/min. The analysis revealed an endothermic weight loss of (ii) 14.17 wt% from room temperature to 63°C, attributed to the evaporation of weakly adsorbed water. Another endothermic weight loss of (iii) 6.46 wt% occurred between 63°C and 144°C, assigned to the evaporation of strongly adsorbed water. Additionally, the TGA indicated a faint endothermic weight loss of (iv) up to approximately 240°C. The ideal formula for the UiO-66 framework is $[Zr_6O_4OH_4(bdc)_6]$, where bdc represents $C_6H_4(COO)_2$. The observed weight losses (i) and (iv) 8.65 wt% suggest a partial replacement of bdc with acetate (ac = CH_3COO)[35], resulting in the

formula [$Zr_6O_4OH_4(14bdc)_{5.28}(ac)_{1.44}$]. To minimize the bdc linker defect, the experimental conditions were carefully adjusted.

X-ray scattering measurements and Rietveld analysis. X-ray diffraction data were collected using a Varex Imaging XRD1621 flat-panel detector in the Debye–Scherrer geometry with monochromatized synchrotron radiation (68.444 keV, λ = 0.18114 Å, calibrated using NIST 674b CeO2 standard) at the BL22XU beamline at SPring-8. The powder samples were enclosed into a Cole–Parmer Kapton capillary (ϕ = 1.0 mm). In particular, the fully and partially dried samples were sealed into the capillary in a glove box, and in order to prevent any unintended water uptake. These two samples were kept under inert gas (N_2) until the X-ray scattering measurements. High-energy X-rays were employed to access high Q-vector regions (0.90–30.4 $Å^{-1}$, 2θ = 1.49–52.0°) and achieve high real-space resolution (0.207 Å). However, their sensitivity to slight changes in lattice parameters might be lower compared to low-energy XRD due to peak overlap and broadening. Additionally, the presence of a direct beamstop protecting the detector from intense synchrotron radiation prevents the observation of low-angle peaks. These drawbacks can sometimes hinder lattice determination, particularly for unknown materials. Therefore, the synthesized UiO-66 product was characterized beforehand. Given the known structure of the UiO-66 framework, we prioritized real-space resolution to accurately determine the positions of water guest molecules within the host framework. Because scattering intensities decrease with scattering angle, to minimize the influence of noise on the analyses even at large diffraction angles, the exposure time was set to 2 second and 150 frames were integrated for each sample. The two-dimensional data were transformed to one-dimensional intensity data using PIXIA in the Orochi suite[3]. The Rietveld and Pawley analysis was carried out with DIFFRAC.TOPAS (version 6) (shown the refinement detail on SI).

Pair distribution function (PDF) analysis. The PDFs and structure factors were obtained from elastic scattering intensity, $I_{elastic}$, extracted from the raw hard X-ray scattering intensity, I_{raw}, by removing the Compton scattering intensity, $I_{Compton}$, and the intensity contributions from air and the sample capillary, $I_{air\ and\ capillary}$.

$$I_{elastic} = I_{raw} - \left(I_{Compton} + I_{air\ and\ capillary}\right) \tag{1}$$

The pair distribution function, *G(r)*, was obtained using a sinuous Fourier transform from the experimentally obtained structure factor, *S(Q)*, in the scattering vector *Q* range of Q_{min}: 1.25 and Q_{max}: 24.0 $Å^{-1}$.

$$G(r) = \frac{2}{\pi}\int_{Q_{min}}^{Q_{max}} Q(S(Q) - 1)sin(Qr)dQ \tag{2}$$

Here, *r* represents interatomic distances between pairs of different atoms in the material. The structure factor, *S(Q)*, was obtained from the elastic X-ray scattering intensity, $I_{elastic}$, using the *N*-body Faber–Ziman model (1, 2, 3, …, *i*, … *N*) of the atomic system.

$$S(Q) = \frac{I_{elastic} - N\{\langle f_i(Q)^2\rangle - \langle f_i(Q)\rangle^2\}}{N\langle f_i(Q)\rangle^2} \tag{3}$$

Here, $f_i(Q)$ is the atomic scattering factor for the ith atom. The expectation values $\langle f_i(Q)^2\rangle$ and $\langle f_i(Q)\rangle^2$ were calculated using the atomic ratio of the material containing *M* elements (1, 2, 3, …, *α*, … *N*). The expectation values were obtained as follows:

$$\begin{cases} \langle f_i(Q)^2 \rangle = \sum_{\alpha}^{M} \left(\frac{N_\alpha}{N} f_\alpha(Q) \right)^2 \\ \langle f_i(Q) \rangle^2 = \left(\sum_{\alpha}^{M} \frac{N_\alpha}{N} f_\alpha(Q) \right)^2 \end{cases} \quad (4).$$

Here, N_α is the atomic ratio of the αth element. The total atomic number, N, was estimated from the experimental data and obtained by the curve fitting of the elastic scattering intensity, $I_{elastic}$. PDF generation and processing was conducted in the Orochi suite.

The PDF by normalization method[4] without using the chemical composition (Figure S3b) was obtained by cosine Fourier transform of differential X-ray diffraction intensity amplified in scattering vector Q. The scattering vector Q range of Q_{min}: 0.8 and Q_{max}: 24.0 Å^{-1} was used for cosine Fourier transform.

$$G_{wo\ chem.\ comp.}(r) = \frac{2}{\pi} \int_{Q_{min}}^{Q_{max}} \frac{d}{dx} \{QI(Q)\} cos(Qr) dQ \quad (5)$$

The differential PDF was obtained by minimizing for the mean squared error (MSE) of PDF between hydrate and partially hydrated UiO-66 in the r-range of 0 to 6.0 Å for the weighted parameter.

$$\mathrm{MSE} = \sum_{r=0}^{6.0} \left[G_{wo\ chem.\ comp.}^{hydrated}(r) - (wheight) \times G_{wo\ chem.\ comp.}^{partially\ hydrated}(r) \right]^2 \quad (6)$$

The r-range was decided based on the diameter of the water octamer, and the minimization was conducted by golden-section search in optimize.golden module in SciPy library in Python.

Ab initio calculations. The geometry of UiO-66, hydrated, partially hydrated, and dehydrated, were optimized with water molecules and the water octamer using linear-scaling density functional theory (DFT) in Quickstep (CP2K)[5]. The linear-scaling DFT calculations were conducted using the TZVP-MOLOPT-GTH basis set for H, O, and C and the DZVP-MOLOPT-GGA-GTH basis set for Zr using the Gaussian Plane Wave (GPW) formalism. The optB88-VdW functional was used for all calculations to evaluate dispersion interactions, including Van der Waals and hydrogen-bonding interactions. The calculations were conducted using linear-scaling self-consistent field methods to reduce the computational cost of the large unit cell calculations. The initial structure of UiO-66 containing the water cluster and molecules was modeled based on the Rietveld analysis. The water octamer structure in vacuum was optimized with TURBOMOLE package. The water octamer was calculated in different level theoretical (DFT (ωB97X-D3 functional), RI-RPA (DFT (PBE0) base), and SCS-MP2) with def2-TZVPP basis set.

The NCI analysis is the method to estimate the strength of interactions from the three-dimensional curvature of electron densities. The non-covalent interactions (NCI) analysis[6] was conducted using Critic2[7]. The horizontal axis of NCI graphs (Figure 5) represents the strength of interactions calculated as $\mathrm{sign}(\lambda_2)\rho$. The ρ is electron densities which can be accessed from cube file as kind of output of ab initio calculations. The λ_2 is value to distinguish repulsive or attractive interactions, and they are mathematically obtained as secondary largest eigenvalue of Hessian matrix of electron densities. The reduced density gradient for vertical axis of NCI analysis is defined using the electron density, ρ, as follows:

$$reduced\ density\ gradient = \frac{1}{2(3\pi^2)^{1/3}} \frac{|\nabla\rho|}{\rho^{4/3}} \quad (7)$$

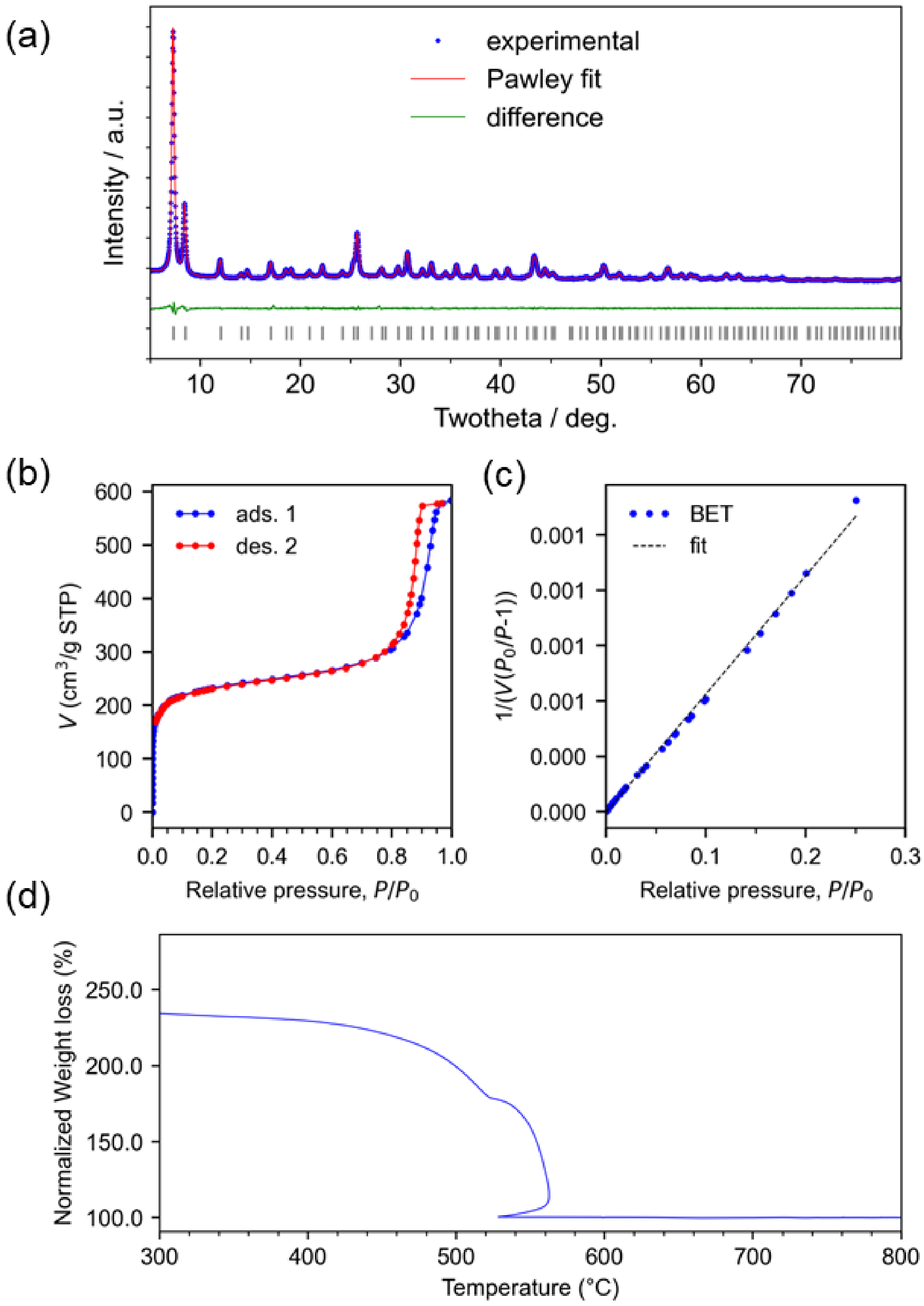

Figure S1 Typical characterization for the synthesized UiO-66 (a) X-ray diffraction measured using Cu Kα for the as-synthesized UiO-66 with the Pawley fitting (space group: $F\bar{4}3m$, a = 20.7587(7) Å, R_{wp} = 3.08%). (b) N_2 adsorption isotherms of UiO-66 (c) BET plot for data of panel “b”. The BET specific surface area is 815.07 m^2/g for the synthesized sample. The BET specific surface area is influenced by missing linker defects in range of 700-1600 m^2/g[8,9]. (d) Normalized thermogravimetric Analysis (TGA) to evaluate defect of bdc linker in UiO-66 as developed in reference[10]. The TGA data was obtained under airflow, and normalized by the weight of plateau region over 600 °C which is assigned as ZrO_2. The observed normalized TGA curve near to the curve for crystal with a few ligand defects reported[10]. The weight loss for linkers was obtained as 48.0 %. whereas, the weight ratio for linkers is 54 % explained as reference[10]. This result shows synthesized UiO-66 with linker (estimated site occupancy: 88%) from TGA analysis.

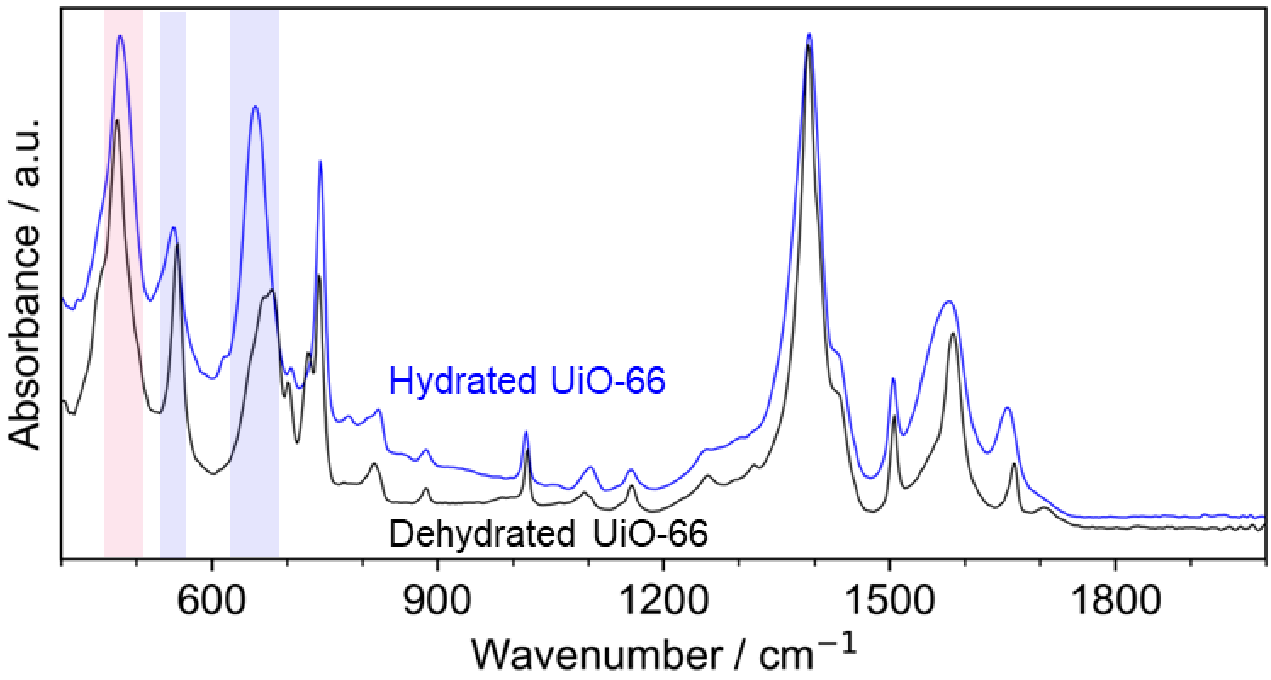

Figure S2 ATR-FTIR spectra for hydrated and dehydrated UiO-66. The vibrational analysis for hydrated UiO-66 (blue line) and dehydrated UiO-66 (black line) samples are investigated. The dehydrated samples are prepared from hydrated samples by removing adsorbed water in evacuation. The water bindings influence UiO-66 vibrations as the blue- and red-shift of peaks (the area highlighted with blue and red color). The peak tops shift from 675, 554, and 474 to 658, 549, and 479 cm^{-1},[10] which correspond to Zr-O vibrations influenced by adsorbed water.

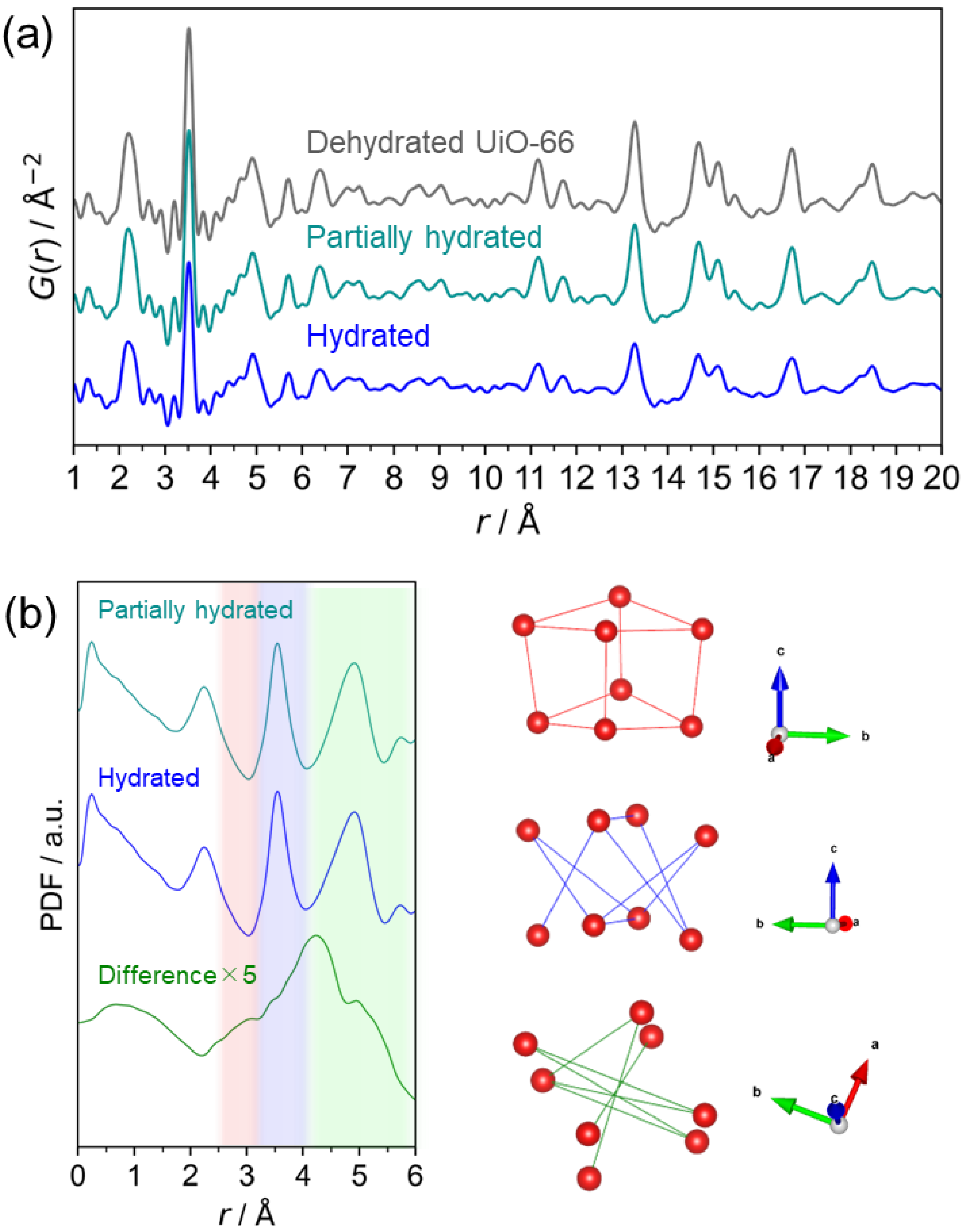

Figure S3 Change of pair distribution functions (PDF) by dehydration of UiO-66. (a) PDF for multistep dehydrations obtained by the Faber-Zimman method using chemical composition $[Zr_6O_4OH_4]$ $[C_6H_4(COO)_2]_6$. (b) PDF for multistep dehydrations obtained by normalization method without using the chemical composition for unclear occupancy of water. The difference PDF was shown with magnified five times for the intensity. The peak in difference PDF between hydrated and partially hydrated phases displays the atomic distance of oxygen-oxygen between adsorbed waters and oxygen-others between adsorbed water and UiO-66. The geometric positions of the oxygen-oxygen atomic pair (the O–O distance for red line: 2.5-3.0 Å, blue line: 3.0-4.0 Å, green line: 4.0-6.0 Å) in water octamer within UiO-66 optimized by DFT calculations was shown in right side. The component unique in water octamer structure was observed, but the peaks assigned to oxygen-zirconium distance (for hydrophobic cavity: 5.1 Å and hydrophilic cavity: 4.3 Å) are dominant in difference PDF.

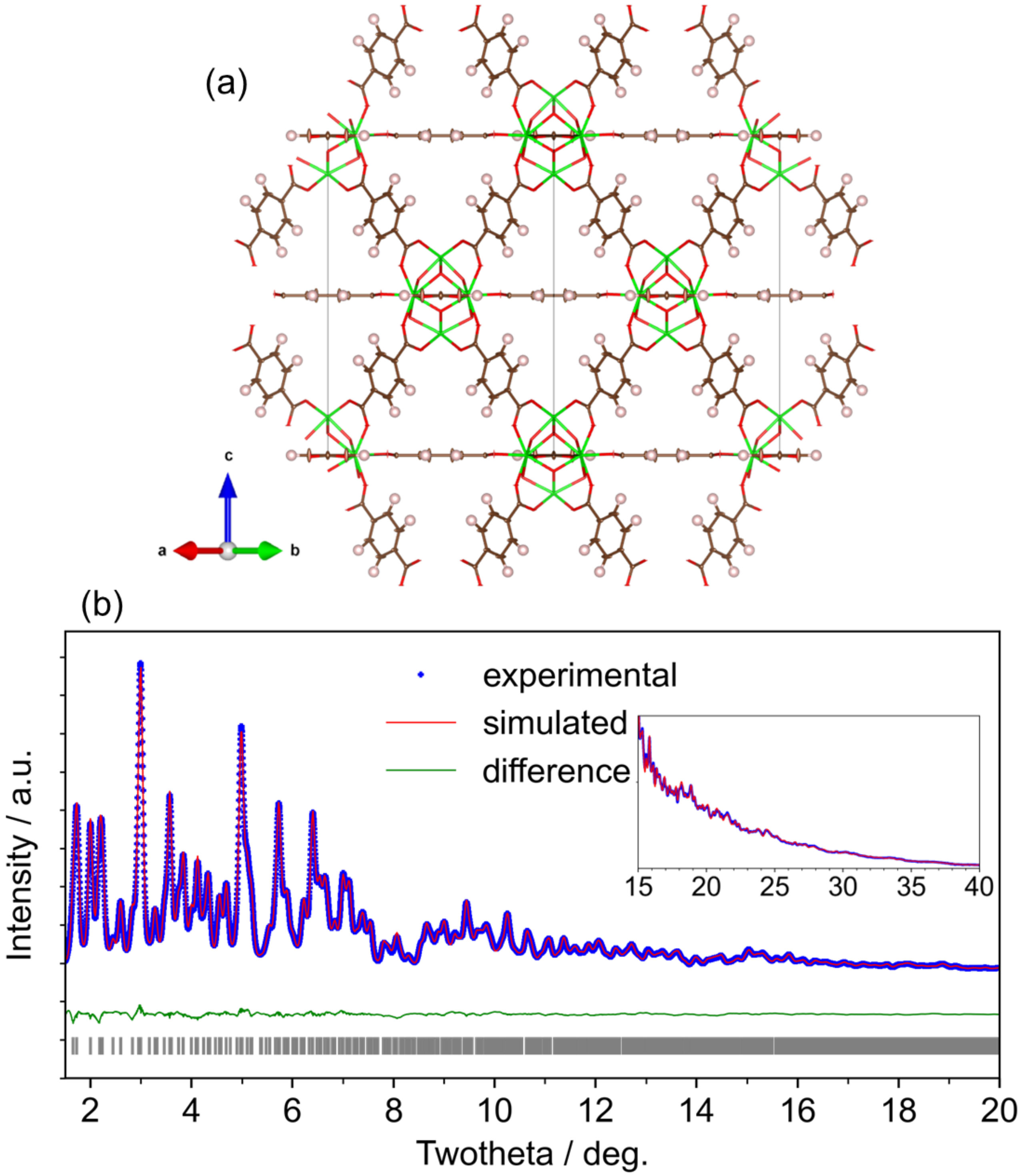

Figure S4 Rietveld fitting for observed XRD pattern of dehydrated UiO-66. (a) Ellipsoidal crystal structural model of dehydrated UiO-66. (b) The fitting results are obtained as cubic phase (space group: $F\bar{4}3m$, a = 20.75497(14) Å, weighted R factor (R_{wp}) = 2.33).

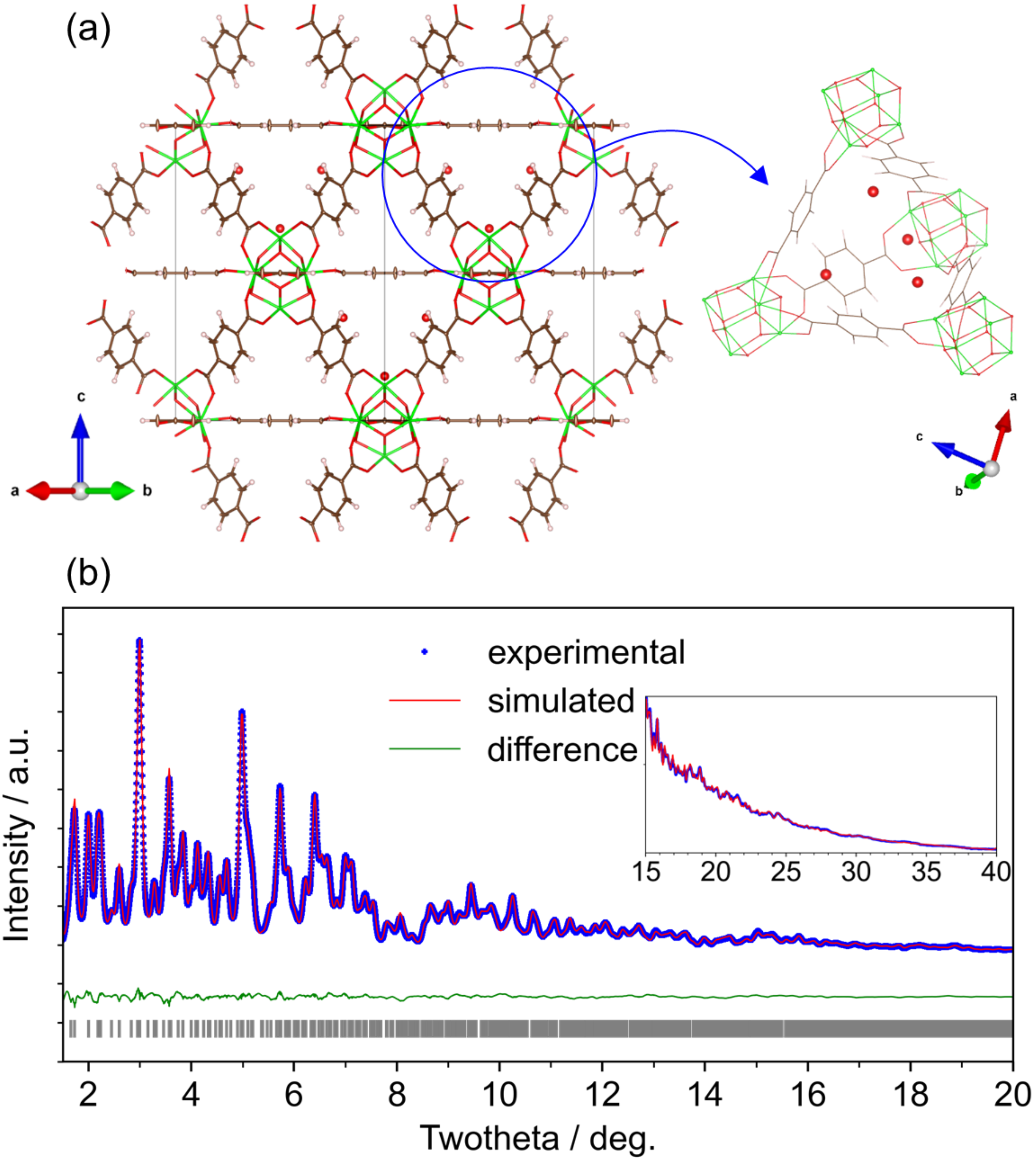

Figure S5 Rietveld fitting for observed XRD pattern of partially hydrated UiO-66. (a) Ellipsoidal crystal structural model of partially hydrated UiO-66. (b) The fitting results are obtained as cubic crystal structure (space group: $F\bar{4}3m$, a = 20.75664(17) Å, R_{wp} = 2.59)

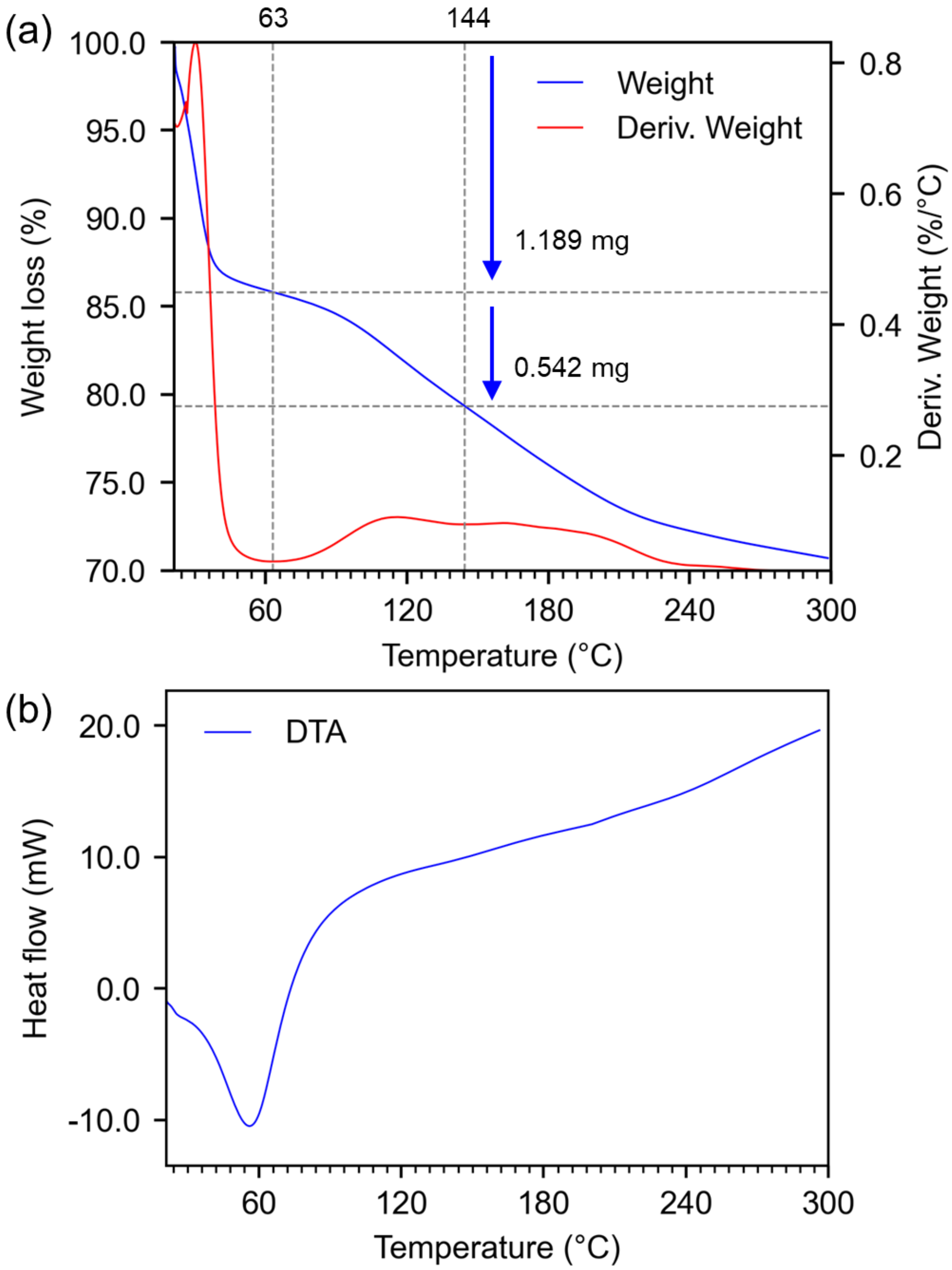

Figure S6 Thermal analysis for adsorbed water in hydrated UiO-66 (a)Thermogravimetric Analysis (TGA) and their derivative (DTG) of hydrated UiO-66 under Argon gas flow (100 ml/sec). The TGA was obtained in range from room temperature to 300 °C. The TGA data below 63 °C shows weight loss for physical adsorption water including water octamer. The TGA data from 63 to 300 °C shows gradual wight loss including multiple components which can be distinguishable by their DTG analysis. The TGA data from 63 to 144 °C are assigned as four adsorbed water molecules in the OH-equipped tetrahedral pores. The weight loss component from 144 to 300 °C can be assigned as dehydration from $Zr_6O_4(OH)_4$ cores explained as reference[10]. (b) The differential Thermal Analysis (DTA) curve of hydrated UiO-66 under Argon gas flow (100 ml/sec) from room temperature to 300 °C.

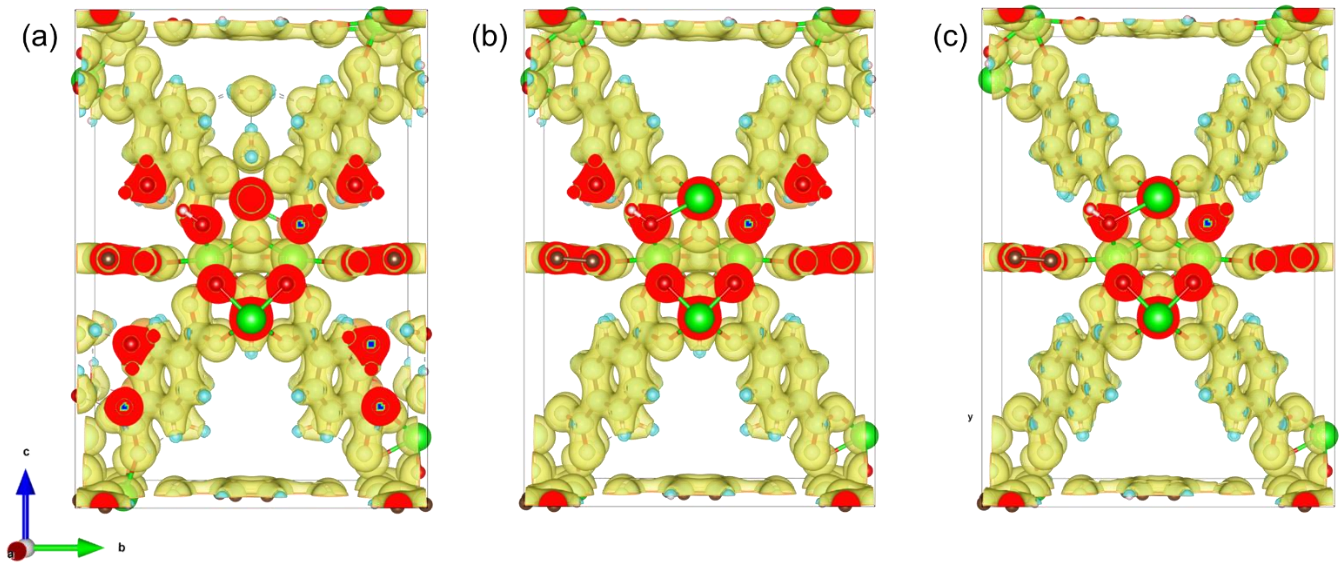

Figure S7 charge density map by DFT calculation for adsorption state of UiO-66, (a) hydrated, (b) partially hydrated, and (c) dehydrated state. The map used to obtain reduced density map in non-covalent interaction analysis.

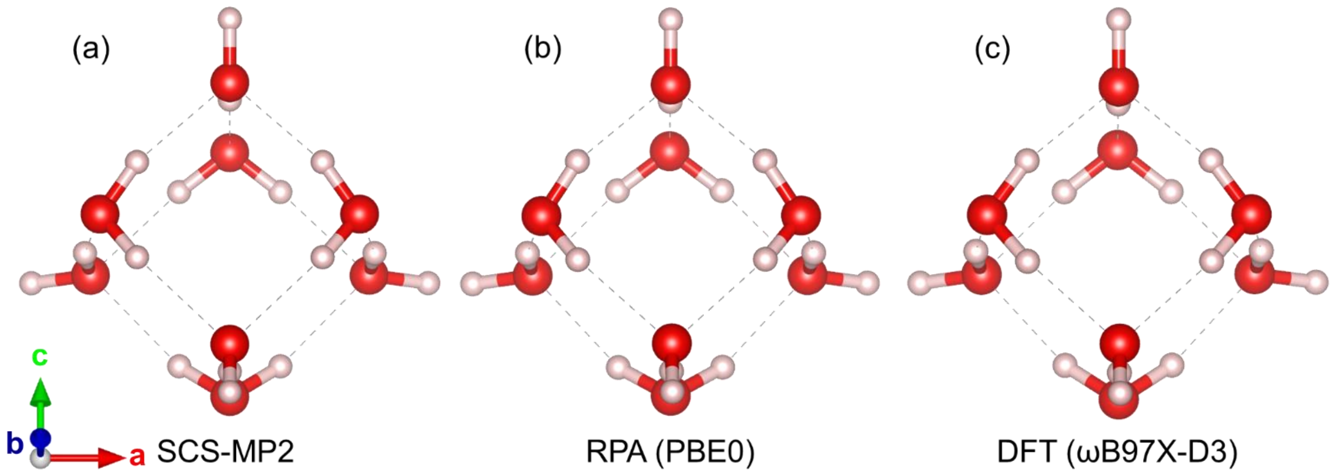

Figure S8 cubane structure by various theoretical methods, (a) DFT (ωB97X-D3), (b) random phase approximation (RPA), and (c) spin-component-scaled second-order Møller-Plesset perturbation theory (SCS-MP2).

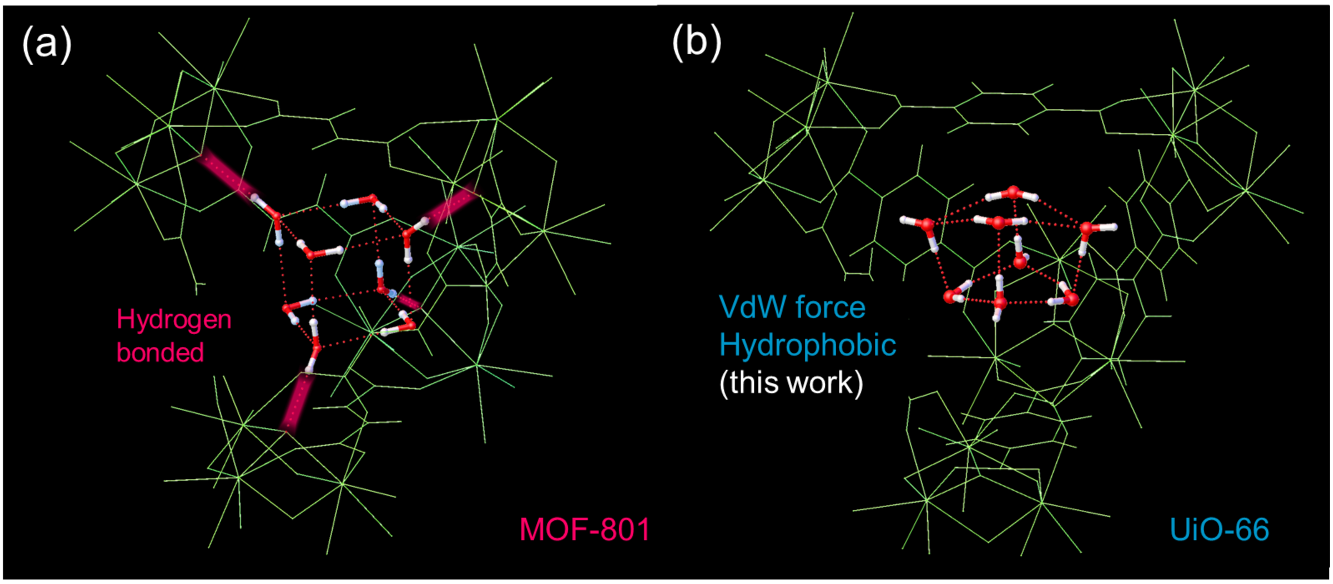

Figure S9 Comparison between the water octamer in (a) MOF-801 connected by hydrogen bonds and (b) UiO-66 stabilized by VdW interactions.

Refinement detail for Rietveld analysis

The Rietveld refinement was conducted in the order of dehydrated, partially hydrated, and hydrated UiO-66 with decreasing space group symmetry to decreasing atomic displacement parameters of adsorbed waters. These investigations sharpen the displacement parameter of water to find the representations as ordered water from disordered conventional representations by finding suitable space groups for Bragg peak changings (Figure 2). The space group symmetry ($F\bar{4}3m$) was suggested by reported works[10], so we started from these space groups for cubic. Additionally, the space group for the whole crystal structure becomes low by the inclusion of water octamer into the crystals, although the symmetry group for only UiO-66 was not changed. The cubane structure of water octamer without structural changes (point group symmetry: D_{2d}) can constructed even using higher symmetry ($F\bar{4}3m$), but the strained water octamer (point group symmetry: C_s) cannot be described on this higher symmetry ($F\bar{4}3m$). The symmetry breaking of the S_4 symmetry of water octamer induces the lowing of the space group symmetry from $F\bar{4}3m$ to their subgroup *Imm*2. Here, the symmetry lowing is shown with Rietveld refinement. The refinement was conducted using DIFFRAC.TOPAS (version 6) for diffraction data (λ = 0.18114 Å) in angle range (2θ = 1.5-40 °).

<u>For the orthorhombic phase space group ($F\bar{4}3m$) without water octamer</u>

The models for this analysis are obtained from the refined model in Figure S11 with removing water octamer. The intensity of the main peaks and lowest angle peaks are mismatched in the Rietveld result. The intensity of the atomic scattering factor for light elements such as oxygen is dominant in low angles, and fitting mismatch owing to the lack of water molecules is remarkable in low angles. The R_{wp} score (R_{wp} = 4.89) becomes also worse compared with the result from the models with water octamers.

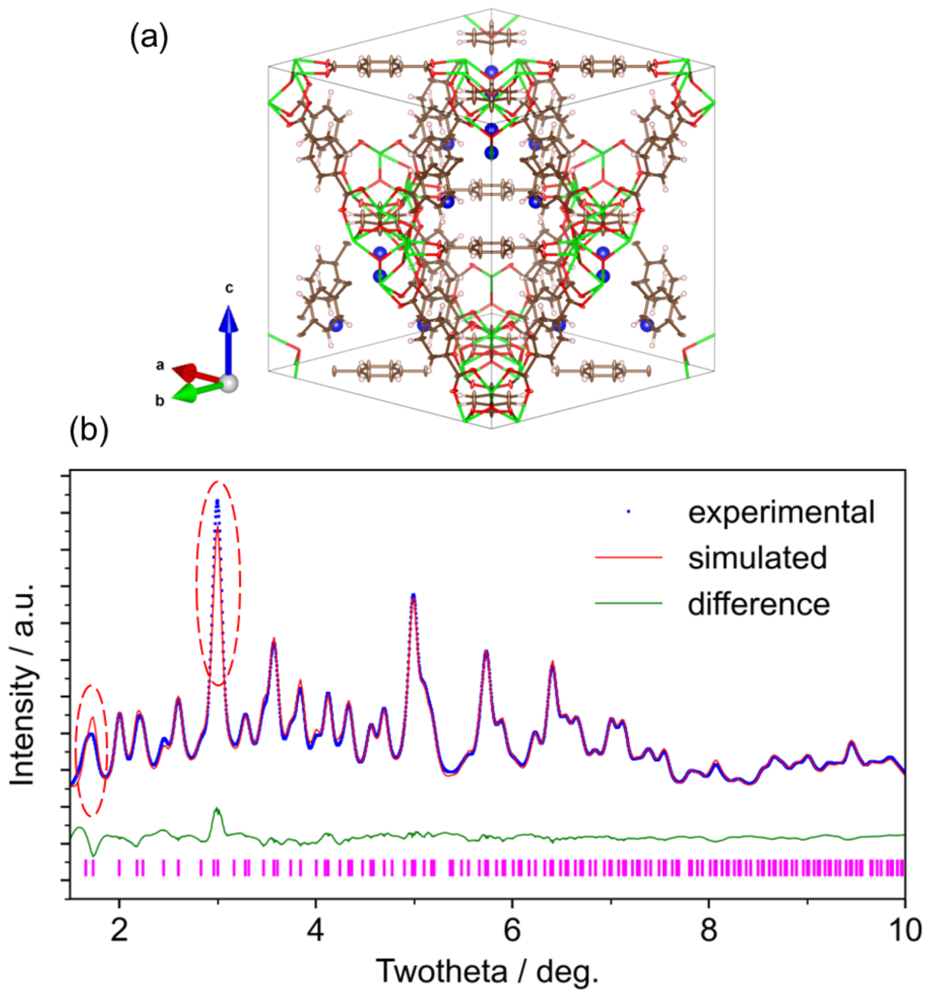

Figure S10 Rietveld analysis for hydrated UiO-66 using partially hydrated UiO-66 model without water octamer. (a) structural model for the Rietveld analysis in panel b. Hydrogen-bonded water positions are shown as blue spheres. (b) The Rietveld analysis for hydrated UiO-66 (R_{wp} = 4.89, space group: $F\bar{4}3m$, a = 20.7413(3) Å). The red circle shows the mismatching of Bragg peaks.

For the cubic phase space group ($F\bar{4}3m$) with dummy atoms for water octamer

The averaged positions of all atoms for UiO-66 with water octamer are refined. The distance restraints (C–H: 1.09 Å) between carbon and hydrogen of phenylene rings were used. The ADP and B_{iso} parameters for all atoms are refined. The occupancy for ligand and water octamer was refined because of ligand defects and hydration levels. The Rietveld fitting results show high correspondence to experimental data with a lower R_{wp} score (R_{wp} = 3.38), but the position displacement for oxygen positions of water octamer becomes large. The average distances (O…O: 2.81 Å) between oxygen atoms of water octamers in this model highly correspond to the average distance (O…O: 2.82 Å) between hydrogen-bonded oxygen of the non-distorted water octamer optimized by the SCS-MP2 method (Figure S8a). The oxygen atoms in water octamer without hydrogen bond interactions with UiO-66 are far from oxygen atoms of the nearest $Zr_6O_4(OH)_4$ core (O…O: 4.56 Å). The displacement of water octamer (B_{iso} = 28.7(19)) was too large to convince crystallographic orders of water octamers, although the eight water molecules seem to present in hydrophobic cages with cubane-like configurations.

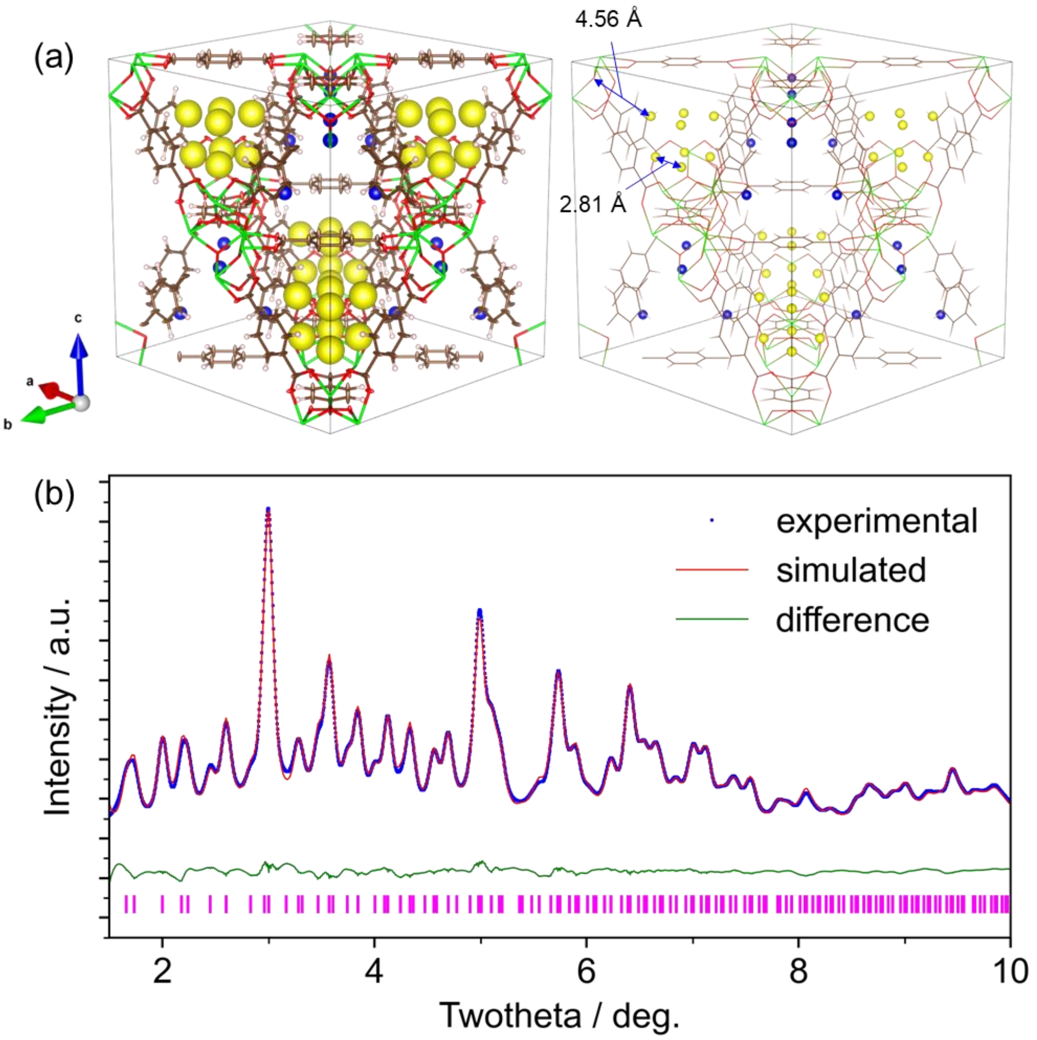

Figure S11 Rietveld analysis for hydrated UiO-66 using hydrated UiO-66 higher symmetry model with water octamer as large displacement parameters (B_{iso} = 28.7(19)). (a) structural model (ellipsoidal and wireframe model) for the Rietveld analysis in panel b. The regions within the ellipsoid represent 50 % probability of the presence of the atom. Hydrogen-bonded water positions are shown as blue spheres. The water positions of the

octamers are shown as yellow spheres. (b) The Rietveld analysis for hydrated UiO-66 (R_{wp} = 3.38, space group: $F\bar{4}3m$, a = 20.7417(3) Å).

For the orthorhombic phase space group (*Imm2*) with water octamer

The averaged positions of $Zr_6O_4(OH)_4$ core and terephthalic acid ligand were fixed to the same positions as the refinement results of Figure S11. The ADP and B_{iso} parameters are refined. The occupancy for ligand and water octamer was refined because of ligand defects and hydration levels. The distance restraint (hydrogen bonded O…O: 2.82 Å) between oxygen atoms of water octamers was used, where their restraint distance was assumed as the averaged distance between hydrogen-bonded oxygen of non-distorted water octamer optimized by the SCS-MP2 method (Figure S8a). The Rietveld fitting results show correspondence with experimental data, and small isotropic displacement for oxygen in water octamers (B_{iso} = 6.3(9)) to convince the presence of water octamers. The water octamer structure is distorted from cubane configurations without distortions while maintaining intra-cluster hydrogen bonds between waters. The slight difference between simulations and experimental data may result from large numbers of optimization parameters in the low space group symmetry model or small rotations or translational moving of water octamers in hydrophobic cages. The displacement parameter of water oxygen bound with hydrogen bond (shown as the blue sphere) displayed smaller atomic displacement parameters (B_{iso} = 2.0(4)) than the displacement of water octamers.

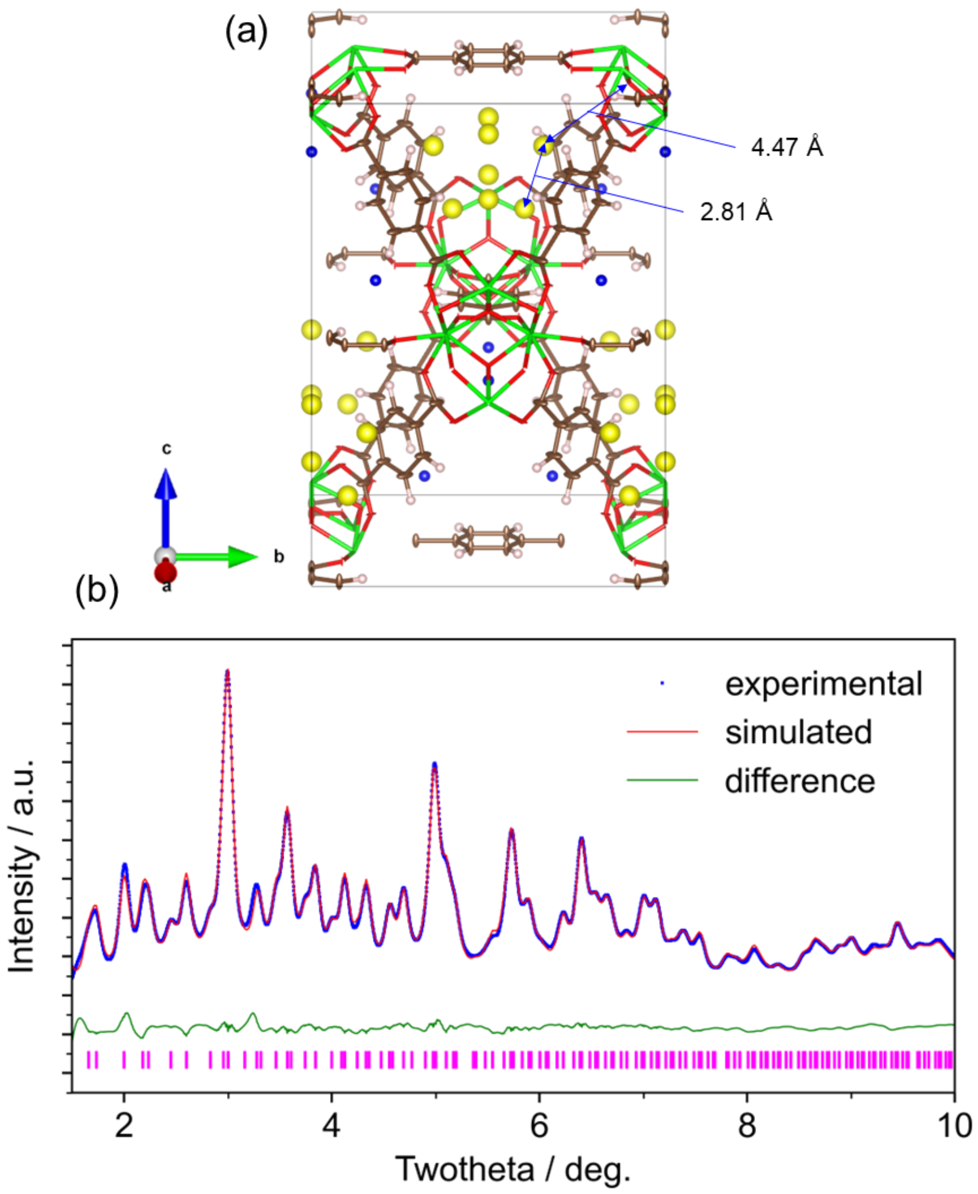

Figure S12 Rietveld analysis for hydrated UiO-66 using hydrated UiO-66 lower symmetry model with water octamer as small displacement parameters (B_{iso} = 6.3(9)). (a) structural model for the Rietveld analysis in panel b. Hydrogen-bonded water positions are shown as blue spheres. The water positions of the octamers are shown as yellow spheres. (b) The Rietveld analysis for hydrated UiO-66 (R_{wp} = 3.82, space group: *Imm*2, *a* = 14.6729(2) Å, *b* = 14.6729(2) Å, *c* = 20.7506(3) Å).

Reference

[51] S. Tominaka, H. Yamada, S. Hiroi, S. I. Kawaguchi, K. Ohara, *ACS Omega* **2018**, *3*, 8874–8881.

[52] K. Sonobe, T. Satoshi, M. Akihiko, *Research Square* **2024**, DOI: 10.21203/rs.3.rs-3930390/v1.

[53] T. D. Kühne, M. Iannuzzi, M. Del Ben, V. V. Rybkin, P. Seewald, F. Stein, T. Laino, R. Z. Khaliullin, O. Schütt, F. Schiffmann, D. Golze, J. Wilhelm, S. Chulkov, M. H. Bani-Hashemian, V. Weber, U. Borštnik, M. Taillefumier, A. S. Jakobovits, A. Lazzaro, H. Pabst, T. Müller, R. Schade, M. Guidon, S. Andermatt, N. Holmberg, G. K. Schenter, A. Hehn, A. Bussy, F. Belleflamme, G. Tabacchi, A. Glöß, M. Lass, I. Bethune, C. J. Mundy, C. Plessl, M. Watkins, J. VandeVondele, M. Krack, J. Hutter, *J. Chem. Phys.* **2020**, *152*, 194103.

[54] A. Otero-de-la-Roza, E. R. Johnson, V. Luaña, *Comput. Phys. Commun.* **2014**, *185*, 1007–1018.

[55] E. R. Johnson, S. Keinan, P. Mori-Sánchez, J. Contreras-García, A. J. Cohen, W. Yang, *J. Am. Chem. Soc.* **2010**, *132*, 6498–6506.

[56] A. Otero-de-la-Roza, E. R. Johnson, V. Luaña, *Comput. Phys. Commun.* **2014**, *185*, 1007–1018.

[57] K. Užarević, T. C. Wang, S. Y. Moon, A. M. Fidelli, J. T. Hupp, O. K. Farha, T. Friščić, *Chem. Commun.* **2016**, *52*, 2133–2136.

[58] L. Valenzano, B. Civalleri, S. Chavan, S. Bordiga, M. H. Nilsen, S. Jakobsen, K. P. Lillerud, C. Lamberti, *Chem. Mater.* **2011**, *23*, 1700–1718.

Structural data

Hydrated UiO-66 (*Imm2*)

```
data_
_chemical_name_mineral UiO-66
_cell_length_a 14.6729(2)
_cell_length_b 14.6729(2)
_cell_length_c 20.7506(3)
_cell_angle_alpha 90
_cell_angle_beta 90
_cell_angle_gamma 90
_cell_volume 4467.46(19)
_symmetry_space_group_name_H-M IMM2
loop_
        _symmetry_equiv_pos_as_xyz
         'x, y, z '
         '-x, y, z '
         'x, -y, z '
         '-x, -y, z '
         'x+1/2, y+1/2, z+1/2 '
         '-x+1/2, y+1/2, z+1/2 '
         'x+1/2, -y+1/2, z+1/2 '
         '-x+1/2, -y+1/2, z+1/2 '
loop_
_atom_site_label
_atom_site_type_symbol
_atom_site_symmetry_multiplicity
_atom_site_fract_x
_atom_site_fract_y
_atom_site_fract_z
_atom_site_occupancy
_atom_site_B_iso_or_equiv
Zr1 Zr   8 0.61962 0.11962 0 1 0.07(2)
Zr2 Zr   2 0.5 0 0.11962 1 0.07(2)
Zr3 Zr   2 0.5 0 0.88038 1 0.07(2)
O1 O   4 0.5 0.8608 0.0696 1 0.00(9)
O2 O   4 0.3608 0 0.9304 1 0.00(9)
O3 O   4 0.5 0.10216 0.94892 1 0.00(9)
O4 O   4 0.60216 0 0.05108 1 0.00(9)
O5 O   8 0.6822 0.8316 0.08959 1 0
O6 O   8 0.3316 0.1822 0.91041 1 0
O7 O   8 0.40351 0.91731 0.1753 1 0
O8 O   8 0.41731 0.90351 0.8247 1 0
O9 O   8 0.41429 0.73511 0.9931 1 0
O10 O   8 0.76489 0.08571 0.0069 1 0
C1 C   8 0.9219 0.0509 0.5 0.768(4) 0
C2 C   8 0.4491 0.5781 0.5 0.768(4) 0
C3 C   4 0.5 0.5934 0 0.768(4) 0
C4 C   4 0.9066 0 0 0.768(4) 0
C5 C   4 0.5 0.7052 0 0.768(4) 0
C6 C   4 0.7948 0 0 0.768(4) 0
C7 C   8 0.7636 0.7364 0.1855 0.768(4) 0
C8 C   8 0.3145 0.8145 0.2636 0.768(4) 0
C9 C   8 0.2364 0.2636 0.8145 0.768(4) 0
C10 C   8 0.3145 0.8145 0.7364 0.768(4) 0
C11 C   8 0.2967 0.7967 0.2033 0.768(4) 0
C12 C   8 0.2967 0.7967 0.7967 0.768(4) 0
C13 C   8 0.3526 0.8526 0.1474 0.768(4) 0
C14 C   8 0.3526 0.8526 0.8526 0.768(4) 0
H1 H   8 0.8568 0.0828 0.5 0.768(4) 1.4(9)
H2 H   8 0.4172 0.6432 0.5 0.768(4) 1.4(9)
H3 H   8 0.363 0.863 0.2802 0.768(4) 1.4(9)
H4 H   8 0.363 0.863 0.7198 0.768(4) 1.4(9)
H5 H   8 0.7802 0.7198 0.137 0.768(4) 1.4(9)
H6 H   8 0.2198 0.2802 0.863 0.768(4) 1.4(9)
W1 O   4 0 0.1808(8) 0.6339(7) 1 2.0(4)
```

```
W2 O   4 0.1808(8) 0 -0.1339(7) 1 2.0(4)
W3 O   4 0 0.156(4) 0.318(4) 1.00(3) 6.3(9)
W4 O   4 0 0.102(3) 0.188(3) 1.00(3) 6.3(9)
W5 O   4 0.1347(18) 0 0.2327(18) 1.00(3) 6.3(9)
W6 O   4 0.091(2) 0 0.3595(18) 1.00(3) 6.3(9)

loop_
_atom_site_aniso_label
_atom_site_type_symbol
_atom_site_aniso_U_11
_atom_site_aniso_U_22
_atom_site_aniso_U_33
_atom_site_aniso_U_12
_atom_site_aniso_U_13
_atom_site_aniso_U_23
O5 O 0.0213(16) 0.0213(16) 0.0010(12) 0.0107(8) 0 0
O6 O 0.0213(16) 0.0213(16) 0.0010(12) 0.0107(8) 0 0
O7 O 0.0213(16) 0.0213(16) 0.0010(12) -0.0107(8) 0 0
O8 O 0.0213(16) 0.0213(16) 0.0010(12) -0.0107(8) 0 0
O9 O 0.0010(12) 0.0010(12) 0.0213(16) 0 0 0
O10 O 0.0010(12) 0.0010(12) 0.0213(16) 0 0 0
C1 C 0.010(2) 0.010(2) 0.055(4) 0 0 0
C2 C 0.010(2) 0.010(2) 0.055(4) 0 0 0
C3 C 0.010(2) 0.010(2) 0.042(5) 0 0 0
C4 C 0.010(2) 0.010(2) 0.042(5) 0 0 0
C5 C 0.010(2) 0.010(2) 0.042(5) 0 0 0
C6 C 0.010(2) 0.010(2) 0.042(5) 0 0 0
C7 C 0.055(4) 0.055(4) 0.010(2) 0.0276(19) 0 0
C8 C 0.055(4) 0.055(4) 0.010(2) -0.0276(19) 0 0
C9 C 0.055(4) 0.055(4) 0.010(2) 0.0276(19) 0 0
C10 C 0.055(4) 0.055(4) 0.010(2) -0.0276(19) 0 0
C11 C 0.042(5) 0.042(5) 0.010(2) -0.021(2) 0 0
C12 C 0.042(5) 0.042(5) 0.010(2) -0.021(2) 0 0
C13 C 0.042(5) 0.042(5) 0.010(2) -0.021(2) 0 0
C14 C 0.042(5) 0.042(5) 0.010(2) -0.021(2) 0 0
```

Hydrated UiO-66 ($F\bar{4}3m$)

```
data_
_chemical_name_mineral UiO-66
_cell_length_a 20.7417(3)
_cell_length_b 20.7417(3)
_cell_length_c 20.7417(3)
_cell_angle_alpha 90
_cell_angle_beta 90
_cell_angle_gamma 90
_cell_volume 8923.5(3)
_symmetry_space_group_name_H-M 216
loop_
        _symmetry_equiv_pos_as_xyz
        'x, y, z '
        '-x, -z, y '
        '-x, z, -y '
        '-x, y, -z '
        '-y, -x, z '
        '-y, -z, x '
        '-y, z, -x '
        '-y, x, -z '
        '-z, -x, y '
        '-z, -y, x '
        '-z, y, -x '
        '-z, x, -y '
        'z, -x, -y '
        'z, -y, -x '
        'z, y, x '
        'z, x, y '
        'y, -x, -z '
        'y, -z, -x '
        'y, z, x '
        'y, x, z '
        'x, -y, -z '
        'x, -z, -y '
        'x, z, y '
        '-x, -y, z '
        'x+1/2, y+1/2, z '
        '-x+1/2, -z+1/2, y '
        '-x+1/2, z+1/2, -y '
        '-x+1/2, y+1/2, -z '
        '-y+1/2, -x+1/2, z '
        '-y+1/2, -z+1/2, x '
        '-y+1/2, z+1/2, -x '
        '-y+1/2, x+1/2, -z '
        '-z+1/2, -x+1/2, y '
        '-z+1/2, -y+1/2, x '
        '-z+1/2, y+1/2, -x '
        '-z+1/2, x+1/2, -y '
        'z+1/2, -x+1/2, -y '
        'z+1/2, -y+1/2, -x '
        'z+1/2, y+1/2, x '
        'z+1/2, x+1/2, y '
        'y+1/2, -x+1/2, -z '
        'y+1/2, -z+1/2, -x '
        'y+1/2, z+1/2, x '
        'y+1/2, x+1/2, z '
        'x+1/2, -y+1/2, -z '
        'x+1/2, -z+1/2, -y '
        'x+1/2, z+1/2, y '
        '-x+1/2, -y+1/2, z '
        'x+1/2, y, z+1/2 '
        '-x+1/2, -z, y+1/2 '
        '-x+1/2, z, -y+1/2 '
        '-x+1/2, y, -z+1/2 '
        '-y+1/2, -x, z+1/2 '
        '-y+1/2, -z, x+1/2 '
```

```
'-y+1/2, z, -x+1/2 '
'-y+1/2, x, -z+1/2 '
'-z+1/2, -x, y+1/2 '
'-z+1/2, -y, x+1/2 '
'-z+1/2, y, -x+1/2 '
'-z+1/2, x, -y+1/2 '
'z+1/2, -x, -y+1/2 '
'z+1/2, -y, -x+1/2 '
'z+1/2, y, x+1/2 '
'z+1/2, x, y+1/2 '
'y+1/2, -x, -z+1/2 '
'y+1/2, -z, -x+1/2 '
'y+1/2, z, x+1/2 '
'y+1/2, x, z+1/2 '
'x+1/2, -y, -z+1/2 '
'x+1/2, -z, -y+1/2 '
'x+1/2, z, y+1/2 '
'-x+1/2, -y, z+1/2 '
'x, y+1/2, z+1/2 '
'-x, -z+1/2, y+1/2 '
'-x, z+1/2, -y+1/2 '
'-x, y+1/2, -z+1/2 '
'-y, -x+1/2, z+1/2 '
'-y, -z+1/2, x+1/2 '
'-y, z+1/2, -x+1/2 '
'-y, x+1/2, -z+1/2 '
'-z, -x+1/2, y+1/2 '
'-z, -y+1/2, x+1/2 '
'-z, y+1/2, -x+1/2 '
'-z, x+1/2, -y+1/2 '
'z, -x+1/2, -y+1/2 '
'z, -y+1/2, -x+1/2 '
'z, y+1/2, x+1/2 '
'z, x+1/2, y+1/2 '
'y, -x+1/2, -z+1/2 '
'y, -z+1/2, -x+1/2 '
'y, z+1/2, x+1/2 '
'y, x+1/2, z+1/2 '
'x, -y+1/2, -z+1/2 '
'x, -z+1/2, -y+1/2 '
'x, z+1/2, y+1/2 '
'-x, -y+1/2, z+1/2 '
loop_
_atom_site_label
_atom_site_type_symbol
_atom_site_symmetry_multiplicity
_atom_site_fract_x
_atom_site_fract_y
_atom_site_fract_z
_atom_site_occupancy
_atom_site_B_iso_or_equiv
Zr Zr  24 0.11962(3) 0 0 1 0.20(2)
O1 O  16 0.06960(16) 0.06960(16) 0.06960(16) 1 0.06(11)
O2 O  16 0.94892(16) 0.94892(16) 0.94892(16) 1 0.06(11)
O3 O  96 0.17530(10) 0.9931(6) 0.08959(10) 1 0
C1 C  48 0.14736(19) 0 0.14736(19) 0.721(5) 0
C2 C  48 0.20328(19) 0 0.20328(19) 0.721(5) 0
C3 C  96 0.2636(3) 0 0.1855(2) 0.721(5) 0
H1 H  96 0.2802(14) 0 0.1371(19) 0.721(5) 1.2(12)
W1 O  16 0.1512(3) 0.1512(3) 0.1512(3) 1.00(2) 7.6(5)
W2 O  16 0.8220(9) 0.8220(9) 0.8220(9) 1.00(5) 28.7(19)
W3 O  16 0.6873(5) 0.6873(5) 0.6873(5) 1.00(5) 28.7(19)

loop_
_atom_site_aniso_label
_atom_site_type_symbol
_atom_site_aniso_U_11
_atom_site_aniso_U_22
```

_atom_site_aniso_U_33
_atom_site_aniso_U_12
_atom_site_aniso_U_13
_atom_site_aniso_U_23
O3 O 0.0112(16) 0.045(5) 0.0100(16) 0.010(3) 0.0000(12) 0.000(3)
C1 C 0.0100(15) 0.071(8) 0.0100(15) 0 0 0
C2 C 0.0100(15) 0.071(9) 0.0100(15) 0 0 0
C3 C 0.0100(15) 0.100(7) 0.0100(15) 0 0 0

Partially hydrated UiO-66

```
data_
_chemical_name_mineral UiO-66
_cell_length_a 20.75664(17)
_cell_length_b 20.75664(17)
_cell_length_c 20.75664(17)
_cell_angle_alpha 90
_cell_angle_beta 90
_cell_angle_gamma 90
_cell_volume 8942.8(2)
_symmetry_space_group_name_H-M 216
loop_
	_symmetry_equiv_pos_as_xyz
	'x, y, z '
	'-x, -z, y '
	'-x, z, -y '
	'-x, y, -z '
	'-y, -x, z '
	'-y, -z, x '
	'-y, z, -x '
	'-y, x, -z '
	'-z, -x, y '
	'-z, -y, x '
	'-z, y, -x '
	'-z, x, -y '
	'z, -x, -y '
	'z, -y, -x '
	'z, y, x '
	'z, x, y '
	'y, -x, -z '
	'y, -z, -x '
	'y, z, x '
	'y, x, z '
	'x, -y, -z '
	'x, -z, -y '
	'x, z, y '
	'-x, -y, z '
	'x+1/2, y+1/2, z '
	'-x+1/2, -z+1/2, y '
	'-x+1/2, z+1/2, -y '
	'-x+1/2, y+1/2, -z '
	'-y+1/2, -x+1/2, z '
	'-y+1/2, -z+1/2, x '
	'-y+1/2, z+1/2, -x '
	'-y+1/2, x+1/2, -z '
	'-z+1/2, -x+1/2, y '
	'-z+1/2, -y+1/2, x '
	'-z+1/2, y+1/2, -x '
	'-z+1/2, x+1/2, -y '
	'z+1/2, -x+1/2, -y '
	'z+1/2, -y+1/2, -x '
	'z+1/2, y+1/2, x '
	'z+1/2, x+1/2, y '
	'y+1/2, -x+1/2, -z '
	'y+1/2, -z+1/2, -x '
	'y+1/2, z+1/2, x '
	'y+1/2, x+1/2, z '
	'x+1/2, -y+1/2, -z '
	'x+1/2, -z+1/2, -y '
	'x+1/2, z+1/2, y '
	'-x+1/2, -y+1/2, z '
	'x+1/2, y, z+1/2 '
	'-x+1/2, -z, y+1/2 '
	'-x+1/2, z, -y+1/2 '
	'-x+1/2, y, -z+1/2 '
	'-y+1/2, -x, z+1/2 '
```

```
        '-y+1/2, -z, x+1/2 '
        '-y+1/2, z, -x+1/2 '
        '-y+1/2, x, -z+1/2 '
        '-z+1/2, -x, y+1/2 '
        '-z+1/2, -y, x+1/2 '
        '-z+1/2, y, -x+1/2 '
        '-z+1/2, x, -y+1/2 '
        'z+1/2, -x, -y+1/2 '
        'z+1/2, -y, -x+1/2 '
        'z+1/2, y, x+1/2 '
        'z+1/2, x, y+1/2 '
        'y+1/2, -x, -z+1/2 '
        'y+1/2, -z, -x+1/2 '
        'y+1/2, z, x+1/2 '
        'y+1/2, x, z+1/2 '
        'x+1/2, -y, -z+1/2 '
        'x+1/2, -z, -y+1/2 '
        'x+1/2, z, y+1/2 '
        '-x+1/2, -y, z+1/2 '
        'x, y+1/2, z+1/2 '
        '-x, -z+1/2, y+1/2 '
        '-x, z+1/2, -y+1/2 '
        '-x, y+1/2, -z+1/2 '
        '-y, -x+1/2, z+1/2 '
        '-y, -z+1/2, x+1/2 '
        '-y, z+1/2, -x+1/2 '
        '-y, x+1/2, -z+1/2 '
        '-z, -x+1/2, y+1/2 '
        '-z, -y+1/2, x+1/2 '
        '-z, y+1/2, -x+1/2 '
        '-z, x+1/2, -y+1/2 '
        'z, -x+1/2, -y+1/2 '
        'z, -y+1/2, -x+1/2 '
        'z, y+1/2, x+1/2 '
        'z, x+1/2, y+1/2 '
        'y, -x+1/2, -z+1/2 '
        'y, -z+1/2, -x+1/2 '
        'y, z+1/2, x+1/2 '
        'y, x+1/2, z+1/2 '
        'x, -y+1/2, -z+1/2 '
        'x, -z+1/2, -y+1/2 '
        'x, z+1/2, y+1/2 '
        '-x, -y+1/2, z+1/2 '
loop_
_atom_site_label
_atom_site_type_symbol
_atom_site_symmetry_multiplicity
_atom_site_fract_x
_atom_site_fract_y
_atom_site_fract_z
_atom_site_occupancy
_atom_site_B_iso_or_equiv
Zr Zr  24 0.119530(16) 0 0 1 0.385(11)
O1 O  16 0.06970(9) 0.06970(9) 0.06970(9) 1 0.21(7)
O2 O  16 0.94884(9) 0.94884(9) 0.94884(9) 1 0.21(7)
O3 O  96 0.17215(5) -0.0094(3) 0.09222(6) 1 0
C1 C  96 0.26732(13) 0 0.18179(11) 0.832(2) 0
C2 C  48 0.15173(9) 0 0.15173(9) 0.832(2) 0
C3 C  48 0.20540(10) 0 0.20540(10) 0.832(2) 0
H1 H  96 0.2919(7) 0 0.1347(10) 0.832(2) 1.3(6)
Ow1 O  16 0.1508(3) 0.1508(3) 0.1508(3) 0.433(6) 3.5(4)

loop_
_atom_site_aniso_label
_atom_site_type_symbol
_atom_site_aniso_U_11
_atom_site_aniso_U_22
_atom_site_aniso_U_33
```

_atom_site_aniso_U_12
_atom_site_aniso_U_13
_atom_site_aniso_U_23
O3 O 0.0010(4) 0.021(3) 0.0010(4) 0 0 0
C1 C 0.0100(8) 0.094(3) 0.0100(8) 0 0 0
C2 C 0.0100(8) 0.026(3) 0.0100(8) 0 0 0
C3 C 0.0100(8) 0.017(2) 0.0100(8) 0 0 0

Dehydrated UiO-66

```
data_
_chemical_name_mineral UiO-66
_cell_length_a 20.75497(14)
_cell_length_b 20.75497(14)
_cell_length_c 20.75497(14)
_cell_angle_alpha 90
_cell_angle_beta 90
_cell_angle_gamma 90
_cell_volume 8940.59(18)
_symmetry_space_group_name_H-M 216
loop_
	_symmetry_equiv_pos_as_xyz
	'x, y, z '
	'-x, -z, y '
	'-x, z, -y '
	'-x, y, -z '
	'-y, -x, z '
	'-y, -z, x '
	'-y, z, -x '
	'-y, x, -z '
	'-z, -x, y '
	'-z, -y, x '
	'-z, y, -x '
	'-z, x, -y '
	'z, -x, -y '
	'z, -y, -x '
	'z, y, x '
	'z, x, y '
	'y, -x, -z '
	'y, -z, -x '
	'y, z, x '
	'y, x, z '
	'x, -y, -z '
	'x, -z, -y '
	'x, z, y '
	'-x, -y, z '
	'x+1/2, y+1/2, z '
	'-x+1/2, -z+1/2, y '
	'-x+1/2, z+1/2, -y '
	'-x+1/2, y+1/2, -z '
	'-y+1/2, -x+1/2, z '
	'-y+1/2, -z+1/2, x '
	'-y+1/2, z+1/2, -x '
	'-y+1/2, x+1/2, -z '
	'-z+1/2, -x+1/2, y '
	'-z+1/2, -y+1/2, x '
	'-z+1/2, y+1/2, -x '
	'-z+1/2, x+1/2, -y '
	'z+1/2, -x+1/2, -y '
	'z+1/2, -y+1/2, -x '
	'z+1/2, y+1/2, x '
	'z+1/2, x+1/2, y '
	'y+1/2, -x+1/2, -z '
	'y+1/2, -z+1/2, -x '
	'y+1/2, z+1/2, x '
	'y+1/2, x+1/2, z '
	'x+1/2, -y+1/2, -z '
	'x+1/2, -z+1/2, -y '
	'x+1/2, z+1/2, y '
	'-x+1/2, -y+1/2, z '
	'x+1/2, y, z+1/2 '
	'-x+1/2, -z, y+1/2 '
	'-x+1/2, z, -y+1/2 '
	'-x+1/2, y, -z+1/2 '
	'-y+1/2, -x, z+1/2 '
```

```
        '-y+1/2, -z, x+1/2 '
        '-y+1/2, z, -x+1/2 '
        '-y+1/2, x, -z+1/2 '
        '-z+1/2, -x, y+1/2 '
        '-z+1/2, -y, x+1/2 '
        '-z+1/2, y, -x+1/2 '
        '-z+1/2, x, -y+1/2 '
        'z+1/2, -x, -y+1/2 '
        'z+1/2, -y, -x+1/2 '
        'z+1/2, y, x+1/2 '
        'z+1/2, x, y+1/2 '
        'y+1/2, -x, -z+1/2 '
        'y+1/2, -z, -x+1/2 '
        'y+1/2, z, x+1/2 '
        'y+1/2, x, z+1/2 '
        'x+1/2, -y, -z+1/2 '
        'x+1/2, -z, -y+1/2 '
        'x+1/2, z, y+1/2 '
        '-x+1/2, -y, z+1/2 '
        'x, y+1/2, z+1/2 '
        '-x, -z+1/2, y+1/2 '
        '-x, z+1/2, -y+1/2 '
        '-x, y+1/2, -z+1/2 '
        '-y, -x+1/2, z+1/2 '
        '-y, -z+1/2, x+1/2 '
        '-y, z+1/2, -x+1/2 '
        '-y, x+1/2, -z+1/2 '
        '-z, -x+1/2, y+1/2 '
        '-z, -y+1/2, x+1/2 '
        '-z, y+1/2, -x+1/2 '
        '-z, x+1/2, -y+1/2 '
        'z, -x+1/2, -y+1/2 '
        'z, -y+1/2, -x+1/2 '
        'z, y+1/2, x+1/2 '
        'z, x+1/2, y+1/2 '
        'y, -x+1/2, -z+1/2 '
        'y, -z+1/2, -x+1/2 '
        'y, z+1/2, x+1/2 '
        'y, x+1/2, z+1/2 '
        'x, -y+1/2, -z+1/2 '
        'x, -z+1/2, -y+1/2 '
        'x, z+1/2, y+1/2 '
        '-x, -y+1/2, z+1/2 '
loop_
_atom_site_label
_atom_site_type_symbol
_atom_site_symmetry_multiplicity
_atom_site_fract_x
_atom_site_fract_y
_atom_site_fract_z
_atom_site_occupancy
_atom_site_B_iso_or_equiv
Zr Zr  24 0.119656(13) 0 0 1 0.233(9)
O1 O  16 0.06908(7) 0.06908(7) 0.06908(7) 1 0.01(5)
O2 O  16 0.95040(7) 0.95040(7) 0.95040(7) 1 0.01(5)
O3 O  96 0.17097(4) -0.0043(5) 0.09346(5) 1 0
C1 C  96 0.26552(10) 0 0.18317(9) 0.876(2) 0
C2 C  48 0.15176(8) 0 0.15176(8) 0.876(2) 0
C3 C  48 0.20419(7) 0 0.20419(7) 0.876(2) 0
H1 H  96 0.2966(6) 0 0.1356(9) 0.876(2) 4.5(6)

loop_
_atom_site_aniso_label
_atom_site_type_symbol
_atom_site_aniso_U_11
_atom_site_aniso_U_22
_atom_site_aniso_U_33
_atom_site_aniso_U_12
```

_atom_site_aniso_U_13
_atom_site_aniso_U_23
O3 O 0.0010(3) 0.035(2) 0.0010(3) 0 0 0
C1 C 0.0100(6) 0.100(3) 0.0100(6) 0 0 0
C2 C 0.0100(6) 0.0176(18) 0.0100(6) 0 0 0
C3 C 0.0100(6) 0.040(2) 0.0100(6) 0 0 0

Hydrated UiO-66 (DFT optimized)


```
data_
_chemical_name_mineral UiO-66
_cell_length_a                14.674200
_cell_length_b                14.674200
_cell_length_c                20.752399
_cell_angle_alpha             90.000000
_cell_angle_beta              90.000000
_cell_angle_gamma             90.000000

_symmetry_space_group_name_H-M    'P 1'
_symmetry_Int_Tables_number       1

loop_
_symmetry_equiv_pos_as_xyz
   'x, y, z'

loop_
   _atom_site_label
   _atom_site_fract_x
   _atom_site_fract_y
   _atom_site_fract_z
   Zr   -0.380045    0.119898   -0.000031
   Zr    0.379926   -0.120348   -0.000446
   Zr   -0.380016   -0.119841   -0.000194
   Zr    0.379947    0.119443    0.000101
   Zr    0.119985   -0.380092    0.499724
   Zr   -0.120065    0.379984    0.499684
   Zr    0.119977    0.379867    0.499626
   Zr   -0.119985   -0.379926    0.499586
   Zr    0.499994   -0.000339    0.120201
   Zr    0.000021    0.499967   -0.379983
   Zr   -0.499869   -0.000012   -0.120387
   Zr   -0.000070    0.499980    0.379484
   H     0.353631    0.414339    0.000049
   H    -0.353751   -0.414282   -0.000688
   H     0.353695   -0.414218   -0.000802
   H    -0.353760    0.414275    0.000170
   H    -0.146290   -0.085719    0.499701
   H     0.146267    0.085664    0.499658
   H    -0.146309    0.085649    0.499692
   H     0.146279   -0.085678    0.499550
   H    -0.085904   -0.146293    0.000934
   H     0.085798    0.146235    0.000933
   H    -0.085877    0.146268    0.000934
   H     0.085791   -0.146289    0.000957
   H    -0.499873    0.297219    0.195172
   H    -0.499958    0.356991    0.132868
   H    -0.499870   -0.297292    0.195061
   H    -0.499989   -0.357118    0.132713
   H     0.000035   -0.202777   -0.304933
   H     0.000017   -0.142984   -0.367276
   H     0.000248    0.202725   -0.304916
   H     0.000096    0.142951   -0.367264
   H     0.357129   -0.499974    0.366680
   H     0.297047   -0.499971    0.304633
   H    -0.357161   -0.499905    0.366674
   H    -0.296968   -0.499961    0.304670
   H    -0.142832   -0.000075   -0.133247
   H    -0.202848   -0.000151   -0.195327
   H     0.142880    0.000108   -0.133270
   H     0.202945    0.000114   -0.195327
   H     0.000799   -0.159434    0.279715
   H     0.004448   -0.222694    0.342596
   H    -0.051627   -0.091803    0.198822
   H     0.054690   -0.094216    0.198484
   H    -0.052302    0.098428    0.198695
```

H	0.053605	0.099372	0.197955
H	0.074879	0.054482	0.353724
H	0.075819	-0.054368	0.353670
H	-0.073352	0.053832	0.355260
H	-0.073529	-0.054730	0.354966
H	-0.499991	0.191354	0.096663
H	0.499998	-0.193032	0.096617
H	-0.192119	-0.499949	0.403288
H	0.191862	0.499960	0.403325
H	-0.499501	-0.339241	-0.218550
H	0.498478	-0.277851	-0.154964
H	-0.492265	0.277211	-0.158485
H	-0.499692	0.341277	-0.220598
H	-0.446378	0.406839	-0.301386
H	0.447466	0.408019	-0.301745
H	-0.447104	-0.400818	-0.302387
H	0.447120	-0.400664	-0.302521
H	0.374621	0.499608	-0.229851
H	0.305113	0.498579	-0.288307
H	-0.306469	-0.499028	-0.287905
H	-0.374741	-0.499907	-0.229328
H	-0.422718	0.445037	-0.143918
H	-0.422525	-0.445376	-0.143438
H	0.430290	-0.445310	-0.145830
H	0.429646	0.445131	-0.144496
H	-0.000014	0.307274	-0.403365
H	0.000038	-0.307272	-0.403390
H	-0.307981	-0.000030	-0.096685
H	0.308195	-0.000013	-0.096547
H	0.198025	0.001300	0.208482
H	0.129703	0.000470	0.267484
H	0.000252	0.162400	0.280485
H	0.000703	0.222043	0.344983
H	-0.191940	0.000710	0.209876
H	-0.125297	-0.000295	0.270179
H	0.414220	0.353753	-0.499105
H	-0.414204	-0.353732	-0.499019
H	0.414155	-0.353756	-0.499104
H	-0.414149	0.353781	-0.498966
H	-0.218384	0.279398	0.133988
H	0.218276	-0.279441	0.133933
H	-0.218287	-0.279461	0.133893
H	0.218414	0.279363	0.133933
H	0.281696	-0.220586	-0.366184
H	-0.281791	0.220572	-0.365974
H	0.281822	0.220410	-0.365876
H	-0.281722	-0.220554	-0.366107
H	0.365021	0.132695	0.279857
H	-0.365030	-0.132782	0.279731
H	0.364973	-0.132733	0.279747
H	-0.365038	0.132722	0.279873
H	-0.135027	-0.367143	-0.220276
H	0.135135	0.366991	-0.220035
H	-0.134853	0.367371	-0.220320
H	0.134855	-0.367261	-0.220396
H	0.220159	-0.281512	-0.134313
H	-0.219941	0.281557	-0.134147
H	0.220439	0.281440	-0.133970
H	-0.220221	-0.281364	-0.134188
H	-0.279735	0.218562	0.365905
H	0.279809	-0.218551	0.365820
H	-0.279734	-0.218527	0.365784
H	0.279742	0.218595	0.365854
H	0.367450	0.135256	-0.279891
H	-0.367309	-0.135232	-0.280073
H	0.367247	-0.135275	-0.280044
H	-0.367066	0.135154	-0.279802
H	-0.132757	-0.364791	0.220014

H	0.132795	0.364735	0.220034
H	-0.132823	0.364763	0.220054
H	0.132841	-0.364869	0.220035
C	0.416939	0.452885	0.001466
C	-0.417052	-0.452850	0.000868
C	0.416989	-0.452771	0.000857
C	-0.417090	0.452802	0.001481
C	-0.082954	-0.047200	-0.499001
C	0.082931	0.047156	-0.499042
C	-0.082960	0.047138	-0.498988
C	0.082935	-0.047183	-0.499092
C	-0.047214	-0.083001	0.000403
C	0.047120	0.082972	0.000353
C	-0.047200	0.082984	0.000403
C	0.047092	-0.083012	0.000354
C	0.452858	0.417032	-0.499643
C	-0.452869	-0.417015	-0.499559
C	0.452819	-0.417043	-0.499634
C	-0.452815	0.417053	-0.499551
C	0.499857	0.405331	0.000831
C	0.499982	-0.405372	-0.000513
C	-0.000000	-0.094686	0.499733
C	-0.000020	0.094635	0.499799
C	-0.094654	-0.000019	0.001184
C	0.094552	-0.000055	0.001106
C	0.405390	0.499980	-0.498873
C	-0.405400	-0.499968	-0.498724
C	0.499802	0.304228	-0.002880
C	-0.499912	-0.304495	-0.006161
C	0.000001	-0.195601	0.494025
C	-0.000043	0.195556	0.494103
C	-0.195745	-0.000007	0.003602
C	0.195639	-0.000105	0.003617
C	0.304272	0.499968	-0.496431
C	-0.304291	-0.499977	-0.496328
C	-0.231218	0.267247	0.184884
C	0.231147	-0.267337	0.184815
C	-0.231124	-0.267296	0.184782
C	0.231243	0.267261	0.184835
C	0.268822	-0.232724	-0.315292
C	-0.268825	0.232716	-0.315093
C	0.269044	0.232729	-0.315014
C	-0.268918	-0.232737	-0.315208
C	0.314426	0.184080	0.267594
C	-0.314359	-0.184102	0.267494
C	0.314373	-0.184110	0.267512
C	-0.314403	0.184067	0.267624
C	-0.185726	-0.315862	-0.232518
C	0.185919	0.315803	-0.232307
C	-0.185473	0.315990	-0.232507
C	0.185512	-0.315918	-0.232624
C	0.232278	-0.268793	-0.185252
C	-0.232128	0.268855	-0.185067
C	0.232784	0.268639	-0.184880
C	-0.232549	-0.268639	-0.185077
C	-0.267604	0.231269	0.314980
C	0.267618	-0.231314	0.314913
C	-0.267556	-0.231229	0.314860
C	0.267650	0.231288	0.314924
C	0.315942	0.185641	-0.267615
C	-0.315774	-0.185615	-0.267816
C	0.315608	-0.185615	-0.267899
C	-0.315493	0.185566	-0.267652
C	-0.184298	-0.314405	0.232200
C	0.184423	0.314418	0.232207
C	-0.184396	0.314410	0.232269
C	0.184373	-0.314502	0.232241
C	-0.295575	0.201304	0.202531

C	0.295523	-0.201422	0.202426
C	-0.295520	-0.201397	0.202394
C	0.295593	0.201311	0.202508
C	0.204419	-0.298659	-0.297686
C	-0.204456	0.298694	-0.297562
C	0.204576	0.298596	-0.297434
C	-0.204537	-0.298658	-0.297604
C	0.298018	-0.204258	-0.202900
C	-0.297850	0.204255	-0.202672
C	0.298432	0.204013	-0.202538
C	-0.298309	-0.204116	-0.202733
C	-0.201892	0.295816	0.297290
C	0.201925	-0.295891	0.297281
C	-0.201793	-0.295764	0.297211
C	0.201934	0.295835	0.297218
C	-0.343266	0.148369	0.151906
C	0.343240	-0.148602	0.151759
C	-0.343365	-0.148604	0.151781
C	0.343177	0.148376	0.151887
C	0.156762	-0.351500	-0.348335
C	-0.156835	0.351517	-0.348283
C	0.156657	0.351361	-0.348160
C	-0.156674	-0.351486	-0.348291
C	0.350963	-0.156853	-0.152209
C	-0.350813	0.156866	-0.151980
C	0.351092	0.155356	-0.152100
C	-0.351097	-0.155183	-0.152392
C	-0.149043	0.343638	0.347891
C	0.149058	-0.344195	0.347761
C	-0.148973	-0.343489	0.347867
C	0.149060	0.343404	0.347884
O	0.499986	0.136717	0.069110
O	0.499945	-0.138750	0.068766
O	0.000008	-0.361597	-0.431201
O	-0.000030	0.361578	-0.431204
O	0.362825	-0.000317	-0.069038
O	-0.362545	0.000332	-0.069041
O	-0.137601	-0.499997	0.430922
O	0.137288	0.499826	0.430922
O	0.499970	-0.099777	-0.050336
O	-0.499947	0.100075	-0.050306
O	-0.000033	0.400341	0.449634
O	-0.000044	-0.400403	0.449621
O	-0.400102	-0.000184	0.050242
O	0.400090	-0.000417	0.050255
O	0.099768	0.499961	-0.449802
O	-0.099771	-0.499982	-0.449808
O	-0.322017	0.166453	0.093591
O	0.321979	-0.166579	0.093421
O	-0.322376	-0.166608	0.093406
O	0.321817	0.166455	0.093619
O	0.178016	-0.333309	-0.406642
O	-0.178170	0.333291	-0.406564
O	0.177497	0.333166	-0.406542
O	-0.177660	-0.333227	-0.406622
O	0.333369	-0.179010	-0.094022
O	-0.333514	0.179087	-0.093714
O	0.332751	0.173784	-0.093570
O	-0.332401	-0.172886	-0.093736
O	-0.166978	0.322774	0.406227
O	0.167295	-0.325574	0.406322
O	-0.166959	-0.322304	0.406149
O	0.166864	0.321642	0.406108
O	0.400393	0.088310	0.171112
O	-0.400427	-0.088538	0.171118
O	0.400353	-0.088559	0.171095
O	-0.400417	0.088323	0.171132
O	-0.099641	-0.411572	-0.328952

O	0.099637	0.411554	-0.328908
O	-0.099653	0.411564	-0.329012
O	0.099644	-0.411600	-0.329006
O	0.411906	0.099130	-0.171315
O	-0.411970	-0.099358	-0.171567
O	0.411304	-0.099756	-0.171127
O	-0.410979	0.099743	-0.171101
O	-0.088452	-0.400342	0.328840
O	0.088532	0.400318	0.328877
O	-0.088440	0.400357	0.328819
O	0.088304	-0.400425	0.328665
O	0.421705	0.265544	-0.004422
O	-0.421818	-0.265887	-0.009032
O	0.421919	-0.266030	-0.008907
O	-0.421924	0.265759	-0.004232
O	-0.078108	-0.234087	0.491111
O	0.078097	0.234052	0.491134
O	-0.078136	0.234072	0.491104
O	0.078148	-0.234135	0.491059
O	-0.234302	-0.078167	0.004426
O	0.234246	0.078060	0.004399
O	-0.234352	0.078195	0.004323
O	0.234270	-0.078270	0.004434
O	0.265672	0.421784	-0.495589
O	-0.265678	-0.421791	-0.495648
O	0.265713	-0.421858	-0.495627
O	-0.265755	0.421845	-0.495499
O	-0.499997	0.294422	0.148361
O	0.499994	-0.294629	0.148238
O	-0.000004	-0.205434	-0.351763
O	0.000008	0.205401	-0.351741
O	0.294415	-0.499999	0.351412
O	-0.294445	0.499981	0.351455
O	-0.205529	0.000001	-0.148568
O	0.205592	-0.000003	-0.148555
O	0.499990	-0.340928	-0.169685
O	-0.499732	0.340610	-0.171682
O	-0.000363	0.159310	0.329360
O	0.000196	-0.159090	0.328660
O	-0.499853	0.366178	-0.297932
O	-0.499860	-0.359881	-0.296946
O	0.000778	-0.134009	0.203233
O	0.000403	0.140078	0.202896
O	-0.127499	0.000280	0.221326
O	0.133309	0.000165	0.218720
O	0.369839	-0.499945	-0.278607
O	-0.371663	-0.499977	-0.278041
O	0.113284	0.000311	0.346688
O	-0.111613	-0.000402	0.347311
O	-0.385742	0.499893	-0.152543
O	0.392519	0.499934	-0.152376
